# Hidden spontaneous polarisation in the chalcohalide photovoltaic absorber Sn_2_SbS_2_I_3_†

**DOI:** 10.1039/d1mh00764e

**Published:** 2021-07-05

**Authors:** Seán R. Kavanagh, Christopher N. Savory, David O. Scanlon, Aron Walsh

**Affiliations:** Department of Chemistry & Thomas Young Centre, University College London 20 Gordon Street London WC1H 0AJ UK d.scanlon@ucl.ac.uk a.walsh@imperial.ac.uk; Department of Materials & Thomas Young Centre, Imperial College London Exhibition Road London SW7 2AZ UK; Diamond Light Source Ltd., Diamond House, Harwell Science and Innovation Campus Didcot Oxfordshire OX11 0DE UK; Department of Materials Science and Engineering, Yonsei University Seoul 03722 Republic of Korea

## Abstract

Perovskite-inspired materials aim to replicate the optoelectronic performance of lead-halide perovskites, while eliminating issues with stability and toxicity. Chalcohalides of group IV/V elements have attracted attention due to enhanced stability provided by stronger metal-chalcogen bonds, alongside compositional flexibility and ns^2^ lone pair cations – a performance-defining feature of halide perovskites. Following the experimental report of solution-grown tin-antimony sulfoiodide (Sn_2_SbS_2_I_3_) solar cells, with power conversion efficiencies above 4%, we assess the structural and electronic properties of this emerging photovoltaic material. We find that the reported centrosymmetric *Cmcm* crystal structure represents an average over multiple polar *Cmc*2_1_ configurations. The instability is confirmed through a combination of lattice dynamics and molecular dynamics simulations. We predict a large spontaneous polarisation of 37 μC cm^−2^ that could be active for electron–hole separation in operating solar cells. We further assess the radiative efficiency limit of this material, calculating *η*_max_ > 30% for film thicknesses *t* > 0.5 μm.

## Abstract 2

We reveal spontaneous symmetry breaking in an emerging class of mixed-metal chalcohalides, yielding strong lattice polarisation which could aid electron–hole separation in solar photovoltaics.

## Graphical abstract

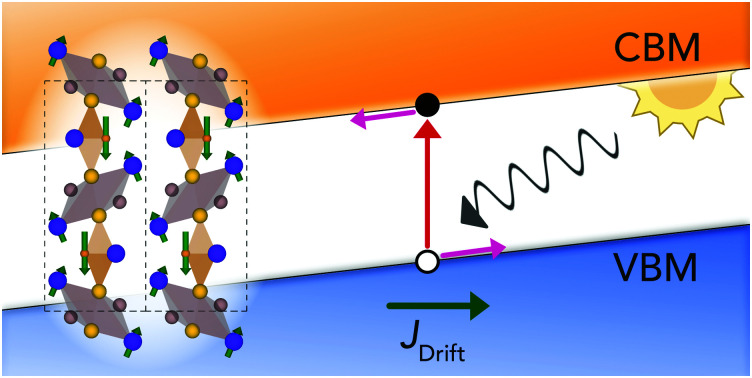

**New concepts**

Mixed-metal chalcohalides have emerged at the forefront of perovskite-inspired materials. A rigorous description of their atomistic properties and performance potential is lacking. In particular, Sn_2_SbS_2_I_3_ contains two lone-pair cations which are known to drive unusual structure–property relations. Using a range of first-principles modelling techniques, we reveal a spontaneous symmetry breaking away from the known non-polar crystal structure. We link this local polarisation, which was previously hidden to macroscopic diffraction techniques, to the high optoelectronic efficiency potential and defect-tolerant properties of this system. The results shine a spotlight on the largely-unexplored class of A_2_BCh_2_X_3_ mixed-metal chalcohalides. These are candidates for solution-processed ferroelectric and optoelectronic devices, with the substitutional flexibility for engineering band gaps, band energies, and lattice polarisation.

The photovoltaic performance of lead-halide perovskites has spurred major research efforts toward the discovery of ‘perovskite-inspired materials’ (PIMs).^1–3^ Through the use of lead-free and stable alternative materials, PIMs aim to replicate the ability of halide perovskites to combine high optoelectronic performance with low-cost solution processing methods, while overcoming their infamous stability and toxicity drawbacks. The applications of PIMs are not solely limited to solar cell devices. In fact, these materials have seen successful implementation in a wide range of optoelectronic applications, such as light-emitting diodes, photocatalysts, radiation detectors, thin film transistors and memristors.^1^

A defining feature of halide perovskites is the combination of a lone-pair cation with a halide anion which can produce, *inter alia*, dispersive valence and conduction bands, defect tolerance, and strong dielectric screening.^1,4^ On the other hand, the soft metal-halide bonding contributes to the poor chemical and thermal stabilities of these materials.^5^ Chalcohalide PIMs offer a route around this issue, demonstrating remarkably higher air and water stability – due to the increased strength of metal-chalcogen bonding^4,5^ – while retaining the ns^2^ cation–halide anion combination. These materials have already demonstrated promising efficiencies (>4%^1,6,7^) alongside low-cost fabrication methods, representing a fertile area for stable, non-toxic high-performance solar cells.^8^ Further increases in power-conversion efficiencies (PCEs) will be required to achieve commercial viability.^5^

Here we focus on tin antimony sulfoiodide (Sn_2_SbS_2_I_3_). Olivier-Fourcade *et al.*^9^ reported the preparation and structural characterisation of Sn_2_SbS_2_I_3_ in 1980, determining an orthorhombic *Cmcm* space group using X-ray diffraction measurements. This work was followed up with single-crystal X-ray analysis by the same research group in 1984.^10^ Later, Dolgikh^11^ prepared both Sn_2_SbS_2_I_3_ and the isostructural, isoelectronic Pb_2_SbS_2_I_3_ compound and investigated the optical dielectric response. Then in 1990, Starosta *et al.*^12^ prepared both antimony sulfoiodide compounds and used photoconductivity measurements to determine optical band gaps of 1.5 and 2.0 eV for the Sn and Pb-based materials, respectively. They also discussed the seemingly anomalous trend of increased band gap with chemical substitution of heavier elements.

In a major breakthrough, Sn_2_SbS_2_I_3_ was recently reported to exhibit a photovoltaic efficiency exceeding 4% in the first experimental device fabrication.^4^ Nie *et al.* used a low-cost single-step deposition method and reported good stability under conditions of high temperature, humidity and illumination. The achievement of a power conversion efficiency exceeding that first reported for methylammonium lead-iodide (MAPI)^13^ is promising. While Sn_2_SbS_2_I_3_ has been known for several decades,^9^ it is now at the forefront of potential defect-tolerant PIMs for optoelectronic applications. In this work, we characterise the physical properties of Sn_2_SbS_2_I_3_ using *ab initio* methods in order to understand the atomistic origins of impressive PV performance, and to assess the efficiency potential going forward.

## Computational methods

We employ a combination of methods to probe the static and dynamic crystal structure of Sn_2_SbS_2_I_3_, in addition to its electronic and optical properties. All base calculations were performed using Density Functional Theory (DFT) within periodic boundary conditions through the Vienna *Ab Initio* Simulation Package (VASP).^14–17^ After testing several DFT exchange–correlation functionals (Section S2.2, ESI†), the optB86b-vdW^18^ functional was chosen for geometry optimisation, yielding the closest agreement with experiment for the *c*/*a* lattice parameter ratio. The ability of this dispersion-corrected functional to incorporate van der Waals interactions in solids has been well demonstrated, yielding accurate predictions of lattice parameters in lone-pair materials.^19–22^ To calculate relative formation energies and the ferroelectric-switching barrier with accuracy beyond DFT, the Random Phase Approximation (RPA) to the correlation energy was employed, using electronic wavefunctions calculated with the HSE06^23^ screened hybrid DFT functional.^24,25^ The HSE06 functional, with full inclusion of spin–orbit coupling effects, was also used for calculations of optical and electronic behaviour – having been demonstrated to yield accurate predictions of band gaps in semiconductor materials.^26,27^ Comprehensive details of the computational implementation are provided in Section S1 of the ESI,† and all calculation data and analyses are provided in an online repository at doi.org/10.5281/zenodo.4683140.

## Structural analysis

**Fig. 1:**
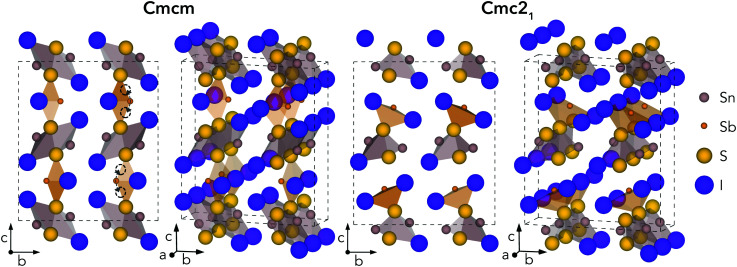
Calculated crystal structures for *Cmcm* and *Cmc*2_1_ polymorphs of Sn_2_SbS_2_I_3_, in the conventional orthorhombic unit cell. Atoms sized according to their formal ionic radii. Curved arrows and empty circles indicate equivalent shifts in Sb position which correspond to the *Cmcm* to *Cmc*2_1_ transition.

Geometry optimisations were performed for Sn_2_SbS_2_I_3_ in both *Cmcm* and *Cmc*2_1_ space groups, using the entries on the Materials Project repository^28^ as the starting points. The relaxed crystal structures and unit cell dimensions are provided in Fig. 1 and Table 1, respectively.

**Table 1:** Calculated lattice parameters of the conventional orthorhombic unit cell for both polymorphs of Sn_2_SbS_2_I_3_, using the optB86b-vdW DFT functional. Experimental values taken from low-temperature (*T* = 173 K) X-ray diffraction measurements^10^

	*c/a*	*a*/Å	*b*/Å	*c*/Å	Vol./Å^3^
*Cmcm*	3.75	4.27	14.02	16.00	957.9
*Cmc*2_1_	3.85	4.29	14.31	16.51	1013.8
Experiment	3.85	4.25	13.99	16.38	973.9

The *Cmcm* structure comprises infinite chains of (Sn_2_S_2_I_2_)_*n*_ along the *a* direction, tightly-packed along the *b* direction to form layers in the *ab* plane, with antimony and iodine atoms located between layers, yielding the overall (Sn_2_SbS_2_I_3_)_*n*_ stoichiometry. The (Sn_2_S_2_I_2_)_*n*_ chains are formed from face-sharing SnS_3_I_2_ pyramids, comprising a parallelepid base of S_2_I_2_ and an apical sulfur atom. In fact, this (Sn_2_S_2_I_2_)_*n*_ structural motif matches the 1D chain structures of the AChX (A = Bi, Sb; Ch = S, Se; X = Br, I) ns^2^-cation chalcohalide family.^29–32^ Moving to the *Cmc*2_1_ crystal structure, the coordination environments of Sb and (to a lesser extent) Sn shift to produce connected chains of the formula unit (Sn_2_SbS_2_I_3_)_*n*_ along the *a* direction, as the Sb atoms attach to the (Sn_2_S_2_I_2_)_*n*_ layers. The *a* and *b* cell lengths are similar for both polymorphs, with the greatest difference occurring along the *c* direction.

Much of the structural behaviour in this system is governed by the lone-pair activities of Sb(iii) and Sn(ii) cations. While the antimony cations are found to exhibit significant localisation of the ns^2^ electrons, only minimal distortion from spherical symmetry is witnessed for the Sn(ii) lone-pair, due to a preferential alignment and thus enhanced interaction with anion p states (Section S3, ESI†). In both polymorphs, the Sb lone-pair is directed toward halide-bordered voids; either *along* the interchain gap (*b* direction) for *Cmcm* symmetry or *toward* the interchain gap (*c* direction) for *Cmc*2_1_ – with more pronounced localisation visible in the *Cmc*2_1_ case (Figs. S6–S8, ESI†). Indeed, this dynamic stereochemical activity of the Sb lone-pair is one of the primary driving factors behind the formation of the distorted, lower-symmetry *Cmc*2_1_ polymorph – through a second-order Jahn–Teller instability, often observed in lone-pair chalcogenides.^22,33–35^

While all experimental works have reported the *Cmcm* crystal structure for Sn_2_SbS_2_I_3_,^9,10,12^ Ibanez *et al.*^10^ noted that assignment of Sb to an 8f Wyckoff position (*i.e.* the Sb Wyckoff site in *Cmc*2_1_ symmetry) with 50% occupancy, as opposed to the 4c site for *Cmcm*, gave a significant reduction in *R*-factor – a measure of agreement between the structure model and diffraction data^36^ – from 0.105 to 0.066. Moreover, both Olivier-Fourcade *et al.*^9^ and Ibanez *et al.*^10^ observed large Debye–Waller (B) displacement factors for the Sb and Sn sites in Sn_2_SbS_2_I_3_ (*i.e.* the site positions which differ most between *Cmcm* and *Cmc*2_1_ structures), even at temperatures as low as *T* = 173 K, alongside large anisotropy in the atomic displacement ellipsoids. Further structural and lone-pair analysis, with direct comparison to experiment (Fig. S4, ESI†), is provided in Sections S2 and S3 (ESI†).

## Thermodynamic & dynamic stability

To ensure a high level of accuracy in the calculated phase stabilities, the Random Phase Approximation (RPA) to the correlation energy was employed. This beyond-DFT method has been demonstrated to yield predictions in excellent agreement with experimental results for the relative formation energies of structural polymorphs.^1,38,39^ With this method, the lower-symmetry *Cmc*2_1_ phase was predicted to be the thermodynamically-favoured polymorph, with a formation energy 35.8 meV per atom below *Cmcm*.

**Fig. 2:**
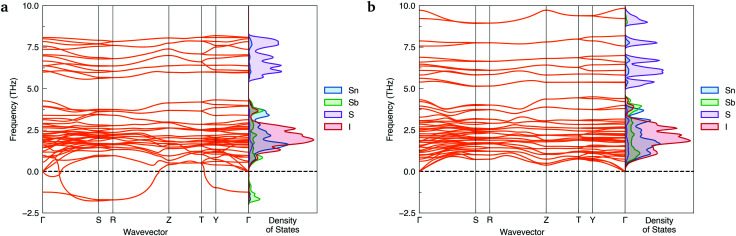
Simulated harmonic phonon dispersions of Sn_2_SbS_2_I_3_ in the *Cmcm* (a) and *Cmc*2_1_ (b) crystal structures, alongside vertical plots of the atom-projected phonon density of states. Generated using ThermoPlotter.^37^ Brillouin zone path shown in Section S10a (ESI†).

Further evidence of *Cmcm* instability was obtained by computing the phonon dispersions of both Sn_2_SbS_2_I_3_ polymorphs – shown in Fig. 2. Imaginary harmonic modes in the phonon dispersion (*i.e.* those with negative frequencies; *ω* < 0) indicate the presence of atomic displacements which lower the system energy. Two strong imaginary modes are witnessed in the *Cmcm* dispersion, demonstrating dynamic structural instability for this polymorph. Using the ISODISTORT^40,41^ package to visualise the imaginary-mode phonon eigenvectors, we confirm that these energy-lowering distortions correspond to different, equivalent *Cmcm* → *Cmc*2_1_ structural transitions (Fig. S5, ESI†).

In contrast, no imaginary modes are observed for the *Cmc*2_1_ polymorph. Thus we find the *Cmc*2_1_ polymorph to exhibit both thermodynamic and dynamic stability, with converse behaviour for the *Cmcm* phase. We propose that experimental reports of *centrosymmetric Cmcm* Sn_2_SbS_2_I_3_ are the result of macroscopic averaging over locally *non-centrosymmetric Cmc*2_1_ configurations. Similar to other ns^2^ cation materials,^22,29,33^ this polar phase behaviour is driven by a second-order (pseudo) Jahn–Teller instability, in which the off-centring of the Sb(iii) ions leads to enhanced bonding interactions between the sp-hybridised Sb ns^2^ lone pair and the anion p states (yielding a small degree of Sb p character at 5 eV below the valence band maximum (VBM); Fig. 5a).

## Spontaneous lattice polarisation

In the *Cmc*2_1_ ground-state structure, the lack of inversion symmetry results in a spontaneous lattice polarisation Δ*P* = 37.0 μC cm^−2^ (calculated within the Berry phase formalism of the Modern Theory of Polarisation).^42^ The strong polarity places Sn_2_SbS_2_I_3_ next to the likes of ferroelectric oxide perovskites such as BaTiO_3_ (∼27 μC cm^−2^)^43^ and KNbO_3_ (∼30 μC cm^−2^), well above that of MAPbI_3_ (‘MAPI’)(4.4 μC cm^−2^)^44^ and the archetypal ‘photoferroic’ SbSI (11 μC cm^−2^).^29,45^ The mirror (*m*) and *c*-glide planes of the *Cmc*2_1_ space group result in zero polarisation in the *ab* plane, with all electronic polarisation directed along the *c* axis, corresponding to the shift in Sb position indicated by the arrows in Fig. 1 and Fig. S5 (ESI†).

We identify substantial mixed ionic-covalent bonding character in this system, reminiscent of that found in ns^2^-cation metal halides.^1,32,46^ Significant covalency is indicated by the cross-band-gap hybridisation of both cation p orbitals with both anion p orbitals, demonstrated by their overlap in both the conduction and valence bands (Fig. 5 and Fig. S13, S22, ESI†). Further evidence of enhanced orbital overlap is derived from the reduced cation–anion distances, relative to their ionic radii, save for the Sb–I pair (Table S1, ESI†). The resulting mixed ionic-covalent bonding gives rise to strong lattice polarisation, with large, anisotropic Born effective charges (Table 2) and dielectric tensor (*ε*_*x*,*y*,*z*_ = [51.3, 18.2, 22.4]).^1,32,47,48^

**Table 2:** Mean Born effective charge tensors (
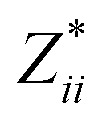
; |*e*|) for each atomic species in Sn_2_SbS_2_I_3_, calculated using the optB86b-vdW DFT functional.^a^ The Born effective charge is a measure of the relationship between polarisation and atomic displacement, and is greater for the *Cmcm* phase due to structural instability.^47,49^ Comparison given to their formal oxidation states

Species	Ox. state	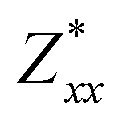	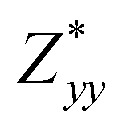	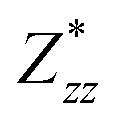
Sn, *Cmcm*	+2	+4.00	+3.00	+3.41
Sn, *Cmc*2_1_	+2	+4.07	+3.16	+2.77
Sb, *Cmcm*	+3	+6.82	+3.55	+9.08
Sb, *Cmc*2_1_	+3	+6.17	+2.84	+6.08
S, *Cmcm*	−2	−2.88	−2.44	−4.40
S, *Cmc*2_1_	−2	−2.69	−2.47	−3.50
I, *Cmcm*	−1	−3.03	−1.55	−2.10
I, *Cmc*2_1_	−1	−2.98	−1.41	−1.53

a Mean Born effective charges were calculated by taking the mean of the diagonal tensor component 
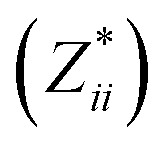
 for each atomic species. Full 
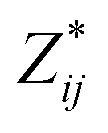
 tensors for each symmetry-inequivalent species are provided in Table S6 (ESI).

**Fig. 3:**
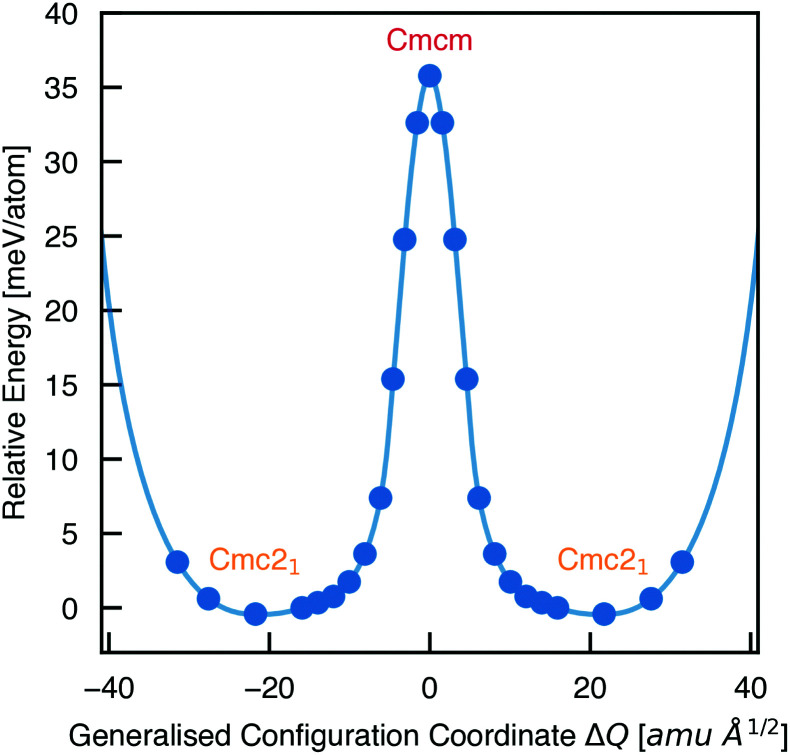
Potential energy surface along the configurational path between equivalent *Cmc*2_1_ configurations of Sn_2_SbS_2_I_3_ (corresponding to the curved arrows in Fig. 1), calculated using the Nudged Elastic Band method.^50^ Filled circles represent calculated data points and the solid line is a spline fit. *X* axis given in units of mass-weighted displacement.

To calculate the barrier to polarisation switching, the Nudged Elastic Band (NEB)^50^ method was employed to map out the potential energy surface (PES) along the minimum-energy path between equivalent *Cmc*2_1_ configurations (Fig. 3).

We find that the *Cmcm* phase corresponds to the transition state between *Cmc*2_1_ configurations, with no local stability around this saddle point on the PES. Consequently, the energetic barrier to ferroelectric switching corresponds to the relative energy of the *Cmcm* and *Cmc*2_1_ polymorphs; Δ*E* = 35.8 meV per atom. This value gives rise to a moderate coercive field – calculated as 750 kV cm^−1^ for a single-crystal ferroelectric domain, using Landau theory (eqn (S1), ESI†).^51^ While the actual value will likely be at least an order of magnitude below this, due to a number of effects including domain formation, it places this material in a range intermediate between the weakly-polar lead-halide perovskites^52^ and the stronger oxide perovskites,^51^ as might be expected for a lone-pair chalcohalide material.

**Fig. 4:**
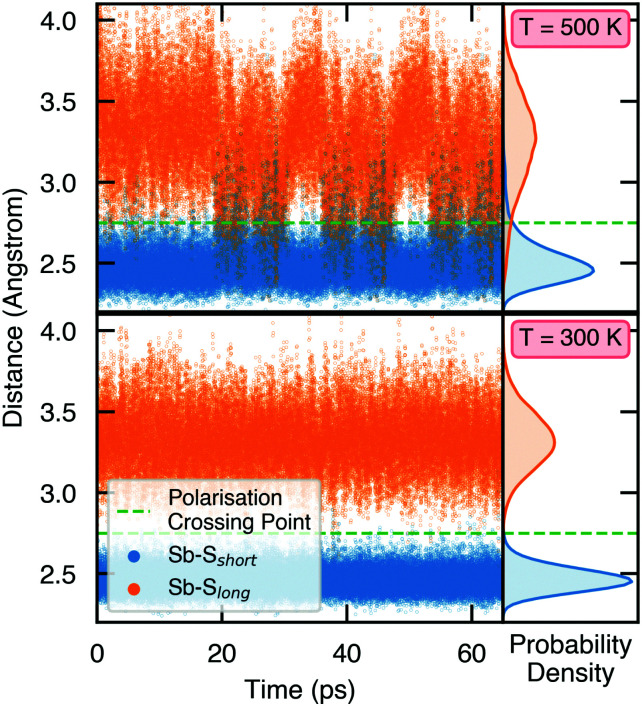
Variation of Sb–S bond lengths in Sn_2_SbS_2_I_3_ as a function of time during molecular dynamics simulations. The probability densities are shown on the right, given by 4π*r*^2^*g*(*r*), where *g*(*r*) is the radial distribution function.

To demonstrate the stability of lattice polarisation at finite temperatures, we performed Molecular Dynamics (MD) simulations for Sn_2_SbS_2_I_3_ within an *NVT* ensemble at temperatures of 300 K and 500 K (Section S8, ESI†). Upon distortion from *Cmcm* to *Cmc*2_1_, the two nearest-neighbour S atoms for Sb become inequivalent, forming short (in the polarisation direction) and long Sb–S bonds (Fig. 1 and Fig. S25, ESI†), allowing the polarisation dynamics to be visualised through the Sb–S bond lengths. As shown in Fig. 4, no appreciable swapping of the Sb–S bonds (corresponding to polarisation switching) is observed for the room-temperature MD runs, within the simulation timescale. On the other hand, transient hopping of Sb atoms occurs during the *T* = 500 K runs, as the material approaches a phase transition to higher-symmetry *Cmcm* (at which point the Sb–S bonds become equivalent and the probability densities merge; Fig. S25, ESI†), indicating a significant decrease in both the strength and stability of lattice polarisation at elevated temperatures. These results confirm the persistence of polar distortions at room temperature and dynamic fluctuations at 500 K. We cannot comment on the size of the polar domains that would be formed, however.

**Fig. 5:**
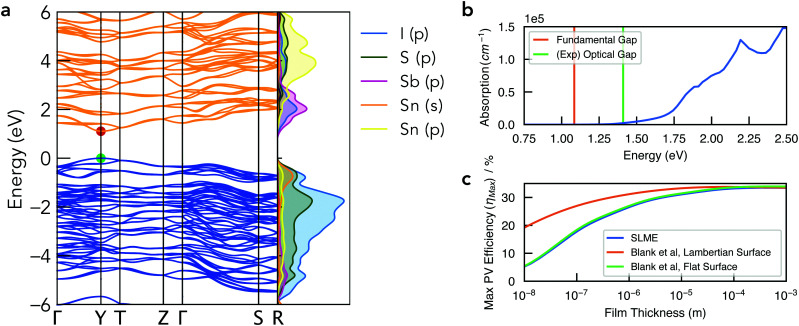
(a) Electronic band structure of Sn_2_SbS_2_I_3_ in the *Cmc*2_1_ crystal structure, alongside a vertical plot of the orbital-projected electronic density of states, generated using sumo.^59^ Valence band in blue, conduction band in orange, and VBM set to 0 eV. Brillouin zone path shown in Fig. S10b (ESI†). (b) Absorption spectrum with vertical lines indicating the calculated electronic band gap and the experimental optical gap (Ref: Nie *et al.*^4^). Note that spectral smearing is expected under experimental measurement. (c) Maximum PV efficiency (*η*_max_) as a function of film thickness, using the SLME^60^ and Blank *et al.*^61^ estimates for the radiative limit (*Q*_*i*_ = 1).

The presence of this previously-hidden polar distortion in Sn_2_SbS_2_I_3_ poses several exciting prospects. In terms of PV applications, spontaneous polarisation can produce open-circuit voltages above the electronic band gap, *via* the Bulk Photovoltaic Effect, potentially allowing efficiencies above the standard limit for a single-junction solar cell.^53–56^ Moreover, the combination of ferroelectric behaviour and spin–orbit coupling could permit switchable spin texture for ‘ferroelectric Rashba semiconductor’ applications,^57^ while the effect on longitudinal optical (LO) phonons could favour polaron formation.^58^

## Electronic structure

The electronic band structure of *Cmc*2_1_ Sn_2_SbS_2_I_3_ is shown in Fig. 4. The band gap is direct, occurring at the Y high-symmetry k-point – which corresponds to maximum antiphase interactions along the conventional *a* crystal direction (*i.e.* along the (Sn_2_SbS_2_I_3_)_*n*_ chains in Fig. 1). The fundamental energy gap is calculated as *E*_g_ = 1.08 eV, placing it in the ideal range for a photovoltaic absorber material, with a ‘detailed-balance’ efficiency limit of 32.5%.^62^

**Fig. 6:**
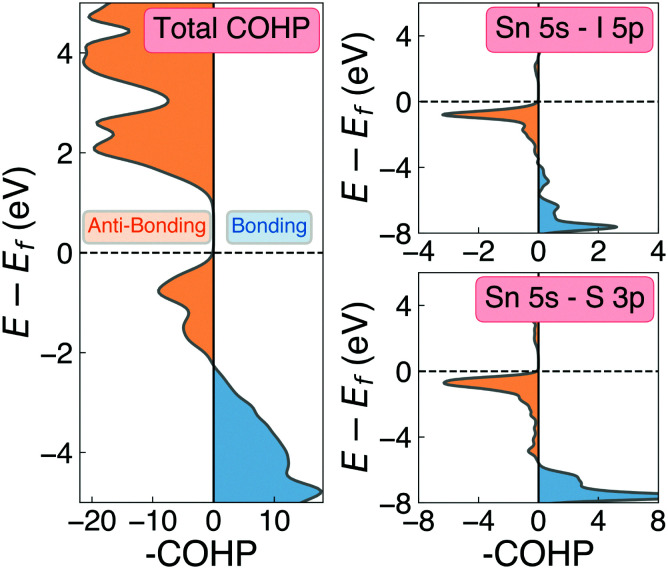
COHP(E) cumulative (left) and Sn 5s – anion p orbital-decomposed (right) analysis of the electronic density of states in Sn_2_SbS_2_I_3_. Negative COHP values (blue) indicate energy-lowering, bonding-type interactions, while positive values (orange) indicate anti-bonding character.

As illustrated by the orbital-projected density of states and band-edge charge densities in Fig. 5a and Fig. S13, S14 (ESI†), the conduction band minimum (CBM) arises from Sb p–I p interactions, while the VBM is comprised of antibonding interactions between the Sn 5s^2^ lone pair and both anion p states (Fig. 6). Notably, this electronic structure allows the explanation of the reported ‘anomalous’ trend in band gap energies within the A_2_SbS_2_I_3_ (A = Sn, Pb) isostructure family, for which Starosta *et al.*^12^ found a gap 0.5 eV *larger* for the Pb-based compound. While band gaps of conventional semiconductors tend to decrease upon substitution of heavier elements, due to increased orbital energies, we witness a strong contradiction to this trend in this material class. This behaviour occurs due to the relativistic contraction of the Pb 6s orbitals, so that the Sn 5s states are in fact higher in energy,^63^ thus enhancing the anti-bonding interaction of the ns^2^ lone-pair with the anion p states at the VBM (Fig. 6).^64^ The result is a more disperse, higher-energy VBM with a reduced energy gap. See Section S6 (ESI†) for a discussion of the effects of spin–orbit interactions and the electronic structure of the *Cmcm* polymorph.

The optical absorption spectrum, shown in Fig. 4, exhibits a weak onset at the fundamental electronic band gap *E*_g_ = 1.08 eV. There are two primary origins of this behaviour. Firstly, we find a low electronic degeneracy at the band extrema (Fig. 5a and Fig. S16, S17, ESI†), itself a consequence of the low crystal symmetry, which rapidly increases with many more interband transitions available at *E* ≥ 1.4 eV. Another contributing factor is a weak transition dipole moment between the VBM and CBM electronic states (Fig. S15–S17, ESI†) – a consequence of both symmetry restriction and low spatial overlap. In the centrosymmetric *Cmcm* structure, the even (*gerade*) parity of both the VBM and CBM wavefunctions about the crystal inversion centre results in a formally symmetry-forbidden transition at the direct gap (as the electric dipole operator is of odd parity, thus yielding an overall zero optical transition matrix element; Section S6, ESI†). For the *Cmc*2_1_ ground-state polymorph, this symmetry selection rule is broken by the shift in Sb positions (removing the inversion symmetry) as well as spin–orbit splitting, however these effects represent a relatively minor perturbation to the electronic structure (Δ*E*_*g*,*Cmcm* *vs.* *Cmc*21_ = 0.03 eV; Fig. S11, ESI†). Consequently, while the direct VBM → CBM transition is no longer formally forbidden in the *Cmc*2_1_ structure, the optical transition matrix element remains weak due to symmetry restraints, compounded by a spatial separation of the VBM and CBM states (Fig. S14, ESI†). The combination of a rapidly increasing joint density of states (JDOS) and optical transition dipole moment (TDM) at energies 0.3–0.5 eV above the direct band gap thus gives rise to a sharp increase in absorption coefficient at *E* ≥ 1.4 eV (Fig. 5b and Fig. S16, ESI†).

This behaviour, we propose, is a likely cause of the apparent mismatch between the calculated fundamental band gap (*E*_g_ = 1.08 eV) and that measured by optical spectroscopy (*E*_Opt,Exp_ = 1.41 eV). While the slow onset of absorption renders the unambiguous determination of an optical gap difficult, Tauc-plot fitting of the calculated absorption within the 1–1.8 eV range gives a best linear fit (with *R*^2^ = 0.93) for an optical gap in the range 1.4–1.5 eV (Fig. S18, ESI†). Indeed, this plot closely resembles that measured by Nie *et al.*,^4^ who also reported a large Urbach energy of 464 meV – which may in part be an artefact of the slow absorption onset.

To quantify the efficiency potential of this material as a photovoltaic (PV) absorber, based on the calculated electronic and optical properties, the maximum PV efficiency (*η*_max_) as a function of film thickness was calculated using both the Spectroscopic Limited Maximum Efficiency (SLME)^60^ and Blank *et al.*^61^ metrics (Fig. 5c). As a consequence of the weak absorption onset, the attainable efficiency shows a strong dependence on film thickness, however this may be combatted through optimisation of the surface scattering properties. Using a Lambertian scattering surface, maximum efficiencies *η*_max_ > 30% are achieved at thicknesses *t* > 0.5 μm, demonstrating the potential application of this material class in high-performance earth-abundant solar cells. Importantly, this model assumes the radiative limit (*i.e.* internal quantum luminescence efficiency *Q*_*i*_ = 1), and so the presence of non-radiative recombination (*Q*_*i*_ < 1) will act to reduce the achievable efficiency and lead to a distinct optimal thickness in the range 0.5–5 μm (Fig. S19, ESI†).

## Potential for defect tolerance

One of the primary origins of non-radiative electron–hole recombination is defects in the bulk crystal and at interfaces. By introducing electronic states within the bandgap, defects can facilitate carrier trapping and annihilation, thus reducing the open-circuit voltage (*V*_oc_) and photovoltaic efficiency.^65–67^ A primary driving factor behind the surge in research interest for ns^2^-cation PIMs is their potential to exhibit defect tolerance – facilitating high efficiencies despite low-cost solution synthesis.^1,68,69^ Sn_2_SbS_2_I_3_ exhibits several material properties which are known to contribute to defect tolerance. Firstly, we find the cation s^2^ and anion p orbitals interact to produce a valence band maximum of anti-bonding character. This bonding behaviour is illustrated by the Crystal Orbital Hamiltonian Population^70,71^ (COHP) analysis shown in Fig. 6, which decomposes the electronic density of states into regions of bonding and anti-bonding orbital interactions. The cation ns^2^-anion p anti-bonding interaction produces a high energy VBM, with an ionisation potential of 5.06 eV (Fig. S9, ESI†) – less than that of MAPI (5.70 eV),^72^ SbSI (5.37 eV)^30^ and Sb_2_Se_3_ (5.13 eV),^35^ and slightly larger than that of FaSnI_3_ (4.88 eV)^73^ – favouring the formation of *shallow* acceptor defects which are innocuous to PV performance. The substantial mixed ionic-covalent character and lattice polarisability in Sn_2_SbS_2_I_3_, discussed previously, results in a strong dielectric screening (*ε*_*x*,*y*,*z*_ = [51.3, 18.2, 22.4]) that will limit the electrostatic interactions between defects and charge carriers, thus reducing the probability of carrier capture and trap-mediated recombination.^1,32^

One-dimensional atomic chain structures, exhibited by this (Fig. 1) and related materials such as Sb_2_Se_3_,^35^ BiOI^32^ and SbSI,^29,30^ can yield benign grain boundaries, greatly reducing charge-carrier recombination in polycrystalline absorber materials.^32,74^ The small electronic band gap (*E*_g_ ∼ 1.1 eV), wide conduction and valence bands, and relatively small electron effective mass (*m*_e_ = 0.29) also favour defect-tolerant behaviour in this material.^1^ Indeed, the presence of moderate defect tolerance is partially suggested by the impressive PV efficiency (*η* > 4%) and photoluminescence lifetimes (>7 ns) obtained by Nie *et al.*^4^ in the first experimental device fabrication for this material.

In conclusion, we present a theoretical characterisation of the Sn_2_SbS_2_I_3_ photovoltaic absorber. While experimental investigations have reported a non-polar, *centrosymmetric Cmcm* crystal structure, we propose that this in fact represents a macroscopic average over multiple *Cmc*2_1_ configurations. Crucially, this leads to the prediction of ferroelectricity, with promising implications for high-efficiency photovoltaic operation and other technological applications.

Through *ab initio* calculation of the electronic and optical properties, we identify an ideal electronic band gap for a photovoltaic absorber (*E*_g_ = 1.08 eV), with power-conversion efficiencies *η*_max_ > 30% at the radiative limit. These features, alongside several properties related to ‘defect tolerance’, present a promising outlook for the potential application of both this material and other unexplored members of the A_2_BCh_2_X_3_ class. Considering only isoelectronic, earth-abundant and non-toxic substituents, there are in fact 36 possible elemental combinations for the quaternary group IV/V chalcohalide family (A = Sn, Ge; B = Sb, Bi; Ch = O, S, Se; X = I, Br, Cl) which may be synthesisable – the majority of which have not yet been investigated.‡‡The substitution of Sn with Pb, Sb with Bi and S with Se has been demonstrated for Sn_2_SbS_2_I_3_.^10–12,75^ Thus the performance potential in this system opens a zoo of compositional permutations for solution-processed ferroelectric and optoelectronic devices.

## Conflicts of interest

There are no conflicts to declare.

## Supplementary Material

MH-008-D1MH00764E-s001
