# Supplementary material for: Hidden spontaneous polarisation in the chalcohalide photovoltaic absorber Sn2SbS2I3
Source: Mater Horiz. 2021 Jul 5;8(10):2709–16. doi: 10.1039/d1mh00764e (PMC8489399; doi:10.1039/d1mh00764e)
Supplement: MH-008-D1MH00764E-s001 [file MH-008-D1MH00764E-s001.pdf]

Electronic Supplementary Material (ESI) for Materials Horizons.
This journal is © The Royal Society of Chemistry 2021

Supplementary Material: Hidden spontaneous polarisation in the chalcohalide photovoltaic absorber $Sn_2SbS_2I_3$

S1 Computational Methods

The `AiiDA` infrastructure was used to maintain data provenance for all calculations performed in this study.[S75] Calculations were performed using Density Functional Theory (DFT) within periodic boundary conditions through the Vienna *Ab Initio* Simulation Package (`VASP`).[S14–S17] Using the projector-augmented wave method, scalar-relativistic potentials were employed to describe the interaction between core and valence electrons.[S76]

S1.1 Structural Relaxation

The optB86b-vdW[S18] exchange-correlation functional was used for geometry optimisation. This choice was motivated by the ability of this dispersion-corrected functional to accurately incorporate van der Waals interactions in solids, yielding accurate predictions of lattice parameters in lone-pair materials.[S19–S22] A convergence criterion of 0.005 eV/Å was imposed on the forces on each atom during structural optimisation. Calculations were carried out with a 14-atom primitive unit cell, using a dense $8\times8\times2$ Γ-centred Monkhorst-Pack **k**-point mesh (equivalent to a **k**-point density of 0.191 Å^{-1} in reciprocal space) and a well-converged 350 eV plane-wave kinetic energy cutoff. For all structural relaxations, the plane-wave energy cutoff was increased to 700 eV in order to avoid the possibility of Pulay stresses.

S1.2 Phase Energetics

To calculate relative formation energies and the ferroelectric-switching barrier with accuracy beyond DFT, the Random Phase Approximation (RPA) to the correlation energy was employed, using electronic wavefunctions calculated with the HSE06[S23] screened hybrid DFT functional.[S24,S25]

S1.3 Electronic Structure & Optical Absorption

The screened hybrid DFT exchange-correlation functional of Heyd, Scuseria and Ernzerhof (HSE06)[S23] was used for calculations of optical and electronic behaviour — having been demonstrated to yield accurate predictions of band gaps in semiconductor materials.[S26,S27] To fully account for relativistic effects - due to the presence of heavy-atom elements Sn, Sb and I, spin–orbit interactions were included (HSE06+SOC) in all electronic and optical calculations. Electronic band structure diagrams and photoemission spectra were generated using the `sumo`[S59] and `Galore`[S77] packages, respectively.

Upper limits to the photovoltaic efficiency were determined from the calculated electronic and optical behaviour, using both the 'Spectroscopic Limited Maximum Efficiency' (SLME) metric proposed by Yu and Zunger[S60] and the method of Blank *et al.*[S61]. The `Irrep`[S78,S79] and `vaspTDM`[S80] packages were used for symmetry analysis of the electronic structure.

Vacuum alignment of the electrostatic potential offset ΔV was performed using the method of Butler *et al.*[S81] with the HSE06+SOC functional, via the `surfaxe` package,[S82] in order to determine the absolute electronic band positions, which were then plotted with `bapt`[S83].

S1.4 Polarisation & Lattice Dynamics

The ionic dielectric response was calculated under Density Functional Perturbation Theory (DFPT),[S84,S85] while the optical response was calculated using the method of Furthmüller *et al.* to obtain the high-frequency real and imaginary dielectric functions.[S84] Lattice polarisation was calculated under the Berry phase formalism of the Modern Theory of Polarisation.[S42] An estimate of the coercive field was obtained using the phenomenological Landau theory,[S51] which gives the following relation between the ferroelectric barrier height ΔE and coercive field E_c:

$$E_c = \frac{4\sqrt{3}}{9}\sqrt{|\chi|\Delta E} \tag{S1}$$

where χ is the electric susceptibility.

The harmonic phonon dispersion was evaluated using the supercell finite-displacement method[S86] within the Phonopy package,[S87] and plotted using the `thermoplotter` package.[S37]

S1.5 *Ab initio* Molecular Dynamics (AIMD)

AIMD simulations were performed using a $3 \times 3 \times 1$ ($24 \times 24 \times 17$ Å) 144-atom supercell expansion of the $Cmc2_1$ primitive unit cell, with the Nosé-Hoover thermostat[S88,S89] in the NVT ensemble and a 2 fs timestep. The same converged plane-wave cutoff energy (350 eV) as the static calculations was used, with $\mathbf{\Gamma}$-point $\mathbf{k}$-space sampling. The PBE-TS[S90] semi-local DFT functional was employed, as this was found to give good agreement with RPA calculations for the polymorph transition energy barrier ($\Delta E_{PBE-TS} = 40.6\,\mathrm{meV/atom}$, $\Delta E_{RPA} = 35.8\,\mathrm{meV/atom}$). For each simulation temperature, volume fitting (using the Birch-Murnaghan equation of state)[S91] followed by temperature equilibration was performed, prior to Molecular Dynamics production runs. `Ovito`[S92] was used to visualise the Molecular Dynamics calculations and the simulation movies are provided as supplementary information and on the Zenodo repository. The `vasppy`[S93] and `DynaPhopy`[S94] packages were used to calculate radial distribution functions and atomic displacement parameters, respectively.

The `pymatgen` package was used throughout for manipulation and analysis of calculation inputs and outputs.[S95] All calculation data and analyses are provided in an online repository at `doi.org/10.5281/zenodo.4683140`.

S2 Supplementary Structural Analysis

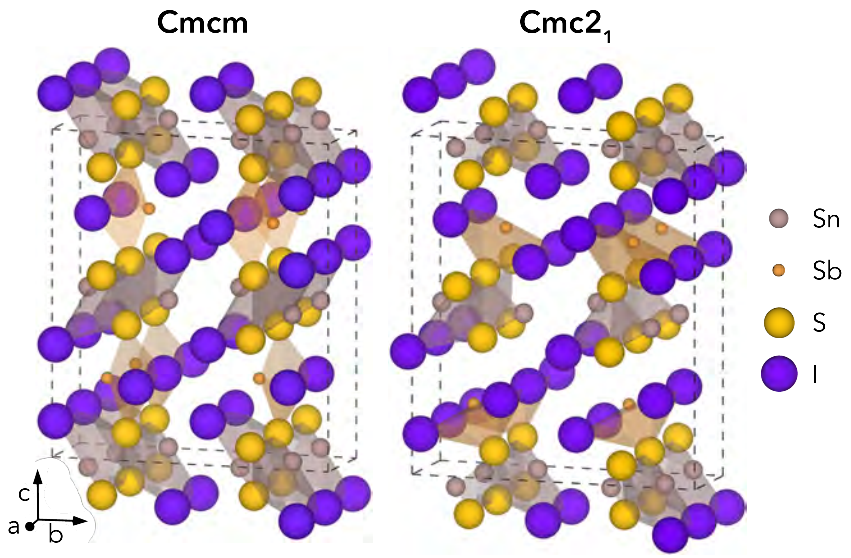

Fig. S1 Calculated crystal structures for $Cmcm$ and $Cmc2_1$ polymorphs of $Sn_2SbS_2I_3$, in the conventional orthorhombic unit cell. Atoms sized according to their formal ionic radii.

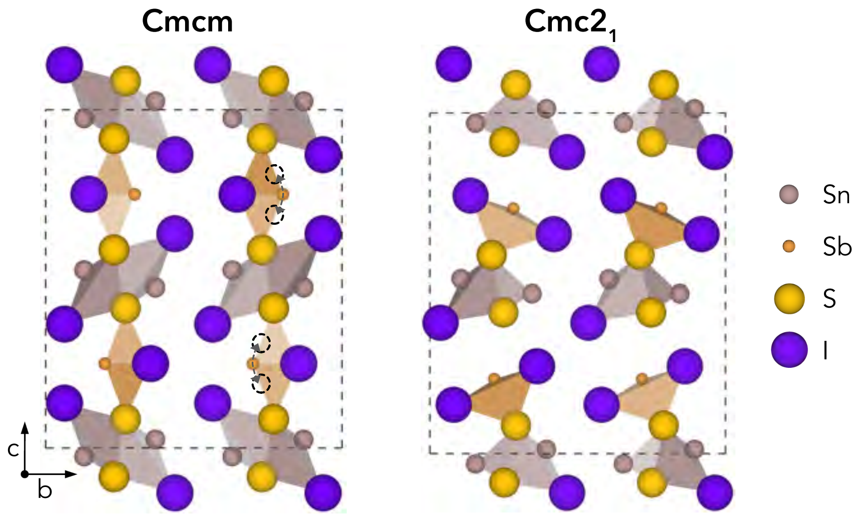

Fig. S2 Calculated crystal structures for $Cmcm$ and $Cmc2_1$ polymorphs of $Sn_2SbS_2I_3$, in the conventional orthorhombic unit cell and projected along the *bc* crystal plane. Atoms sized according to their formal ionic radii.

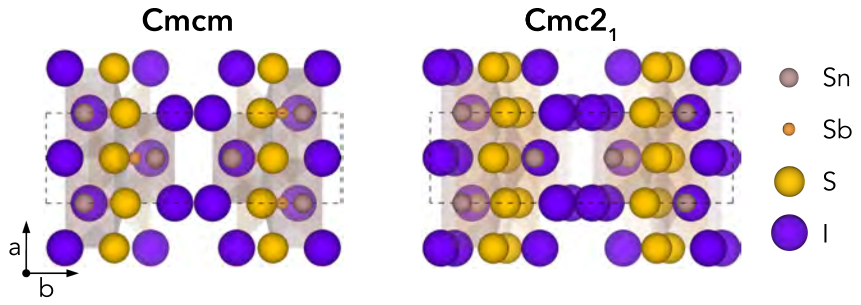

Fig. S3 Calculated crystal structures for *Cmcm* and *Cmc*2$_1$ polymorphs of $Sn_2SbS_2I_3$, in the conventional orthorhombic unit cell and projected along the *ab* crystal plane. Atoms sized according to their formal ionic radii.

In their structural characterisation of $Sn_2SbS_2I_3$ in 1984, Ibanez *et al.* [S10] noted that assignment of Sb to an 8f Wyckoff position (*i.e.* the Sb Wyckoff site in *Cmc*2$_1$ symmetry) with 50% occupancy, as opposed to the 4c site for *Cmcm*, gave a significant reduction in R-factor — a measure of agreement between the structure model and diffraction data[S36] — from 0.105 to 0.066. Both Olivier-Fourcade *et al.* [S9] and Ibanez *et al.* [S10] observed large Debye-Waller (B) displacement factors for the Sb and Sn sites in $Sn_2SbS_2I_3$ (*i.e.* the site positions which differ most between *Cmcm* and *Cmc*2$_1$ structures) even at temperatures as low as T = 173 K, alongside large anisotropy in the 'thermal' displacement ellipsoids.
Moreover, the displacement ellipsoids determined for Sb are indeed directed toward the Sb sites in the *Cmc*2$_1$ structure, with the proposed structure of $Sn_2SbX_2I_3$ (X = S, Se) shown in Fig. S4. This behaviour, the authors propose, is a consequence of the 'loose' coordination environment of Sb (and Sn) with large polarisable anions.

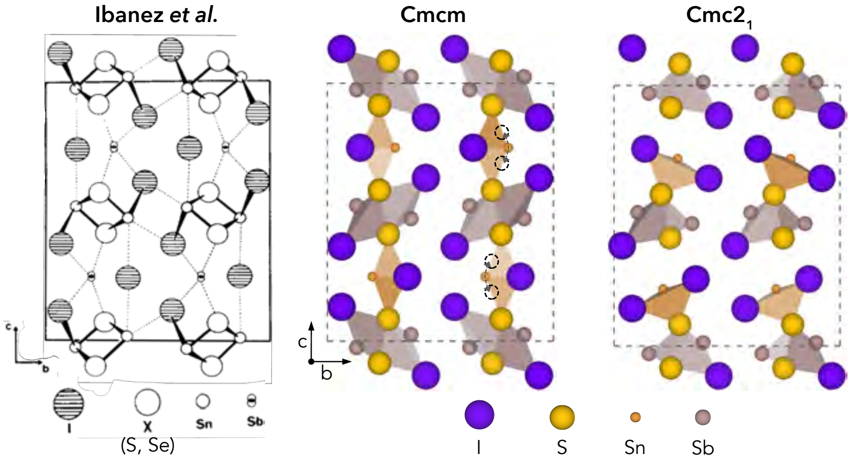

Fig. S4 Experimentally-determined crystal structure of $Sn_2SbX_2I_3$ (X = S, Se), reproduced with permission from Ibanez *et al.* [S10], alongside the calculated crystal structures for both *Cmcm* and *Cmc*2$_1$ polymorphs of $Sn_2SbS_2I_3$. All structures are shown in the conventional orthorhombic unit cell and projected along the *bc* crystal plane. Atoms sized according to their formal ionic radii.

As shown in Fig. S5, the imaginary modes of the *Cmcm* phase, corresponding to a structural transition to the *Cmc*2$_1$ crystal structure, involve small displacements of iodine atoms in the lateral (*ab*) plane — though mostly directed along *c*. However, the atomic displacements along this plane are anti-phase, with equal numbers of iodine atoms moving in the positive and negative directions. The resulting space group ensures no spontaneous polarisation in the *ab* plane. The in-phase displacements of antimony and iodine atoms respectively along the *c* axis, however, remove the (mirror-)plane symmetry (*m*) of the *Cmcm* space group. The outcome is a *Cmc*2$_1$ space group symmetry and a strong electronic polarisation along the crystal *c* axis.

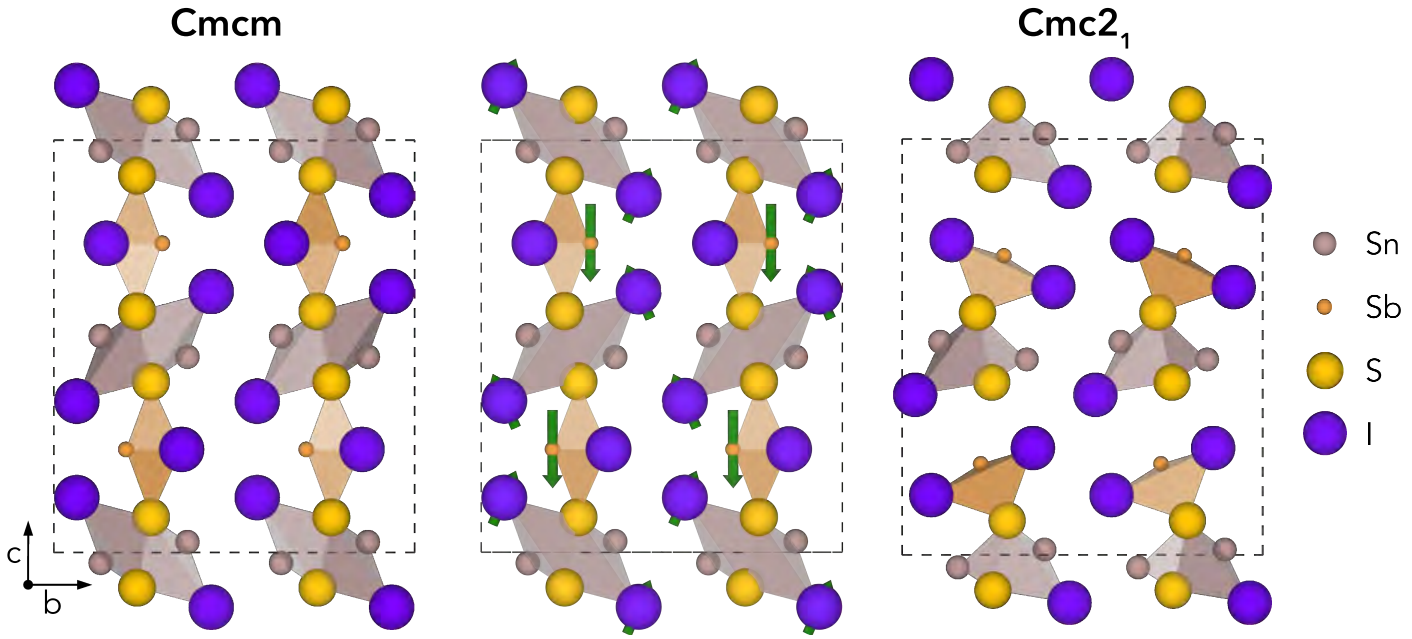

Fig. S5 Vector visualisation of the imaginary phonon mode displacements for the *Cmcm* phase of $Sn_2SbS_2I_3$, confirmed to correspond to a structural transition to the $Cmc2_1$ phase, alongside the calculated crystal structures for both *Cmcm* **(left)** and $Cmc2_1$ **(right)** polymorphs of $Sn_2SbS_2I_3$. The displacements of tin atoms are not visible, as they are significantly smaller than their ionic radii. All structures are shown in the conventional orthorhombic unit cell and projected along the *bc* crystal plane. Atoms sized according to their formal ionic radii.

S2.1 Sb(III) Coordination Environments

In the *Cmcm* arrangement, the Sb atoms adopt a trigonal bipyramidal-like coordination geometry, with two axial iodine ($d^{Cmcm}_{Sb-I(1)} = 3.04\,\text{Å}$), two equatorial sulfur anions ($d^{Cmcm}_{Sb-S} = 2.81\,\text{Å}$) and the Sb lone pair occupying the final equatorial site (Figure S6). The lone pair is directed toward a square planar arrangement of iodine anions, each a distance $d^{Cmcm}_{Sb-I(2)} = 3.42\,\text{Å}$ away from the Sb site.

Transitioning to $Cmc2_1$ symmetry, the Sb atoms shift to a distorted square pyramidal geometry, with two in-plane iodine neighbours at $d^{Cmc2_1}_{Sb-I(1)} = 3.05\,\text{Å}$, the other two at $d^{Cmc2_1}_{Sb-I(2)} = 3.16\,\text{Å}$ and the apical sulfur at $d^{Cmc2_1}_{Sb-S} = 2.49\,\text{Å}$. In this case, the Sb lone pair is directed along the *c* direction, toward the gap between $(Sn_2SbS_2I_3)_n$ chains. In contrast, the Sn^{2+} lone pair shows only minimal distortion from spherical symmetry (Figure S6).

Table S1 Comparison of the *shortest* interatomic distances ('bond lengths') for each cation-anion pair in $Cmc2_1$ $Sn_2SbS_2I_3$, using the optB86b-vdW DFT functional, with the sum of their ionic radii (Σ_{Ionic}). Experimental data for ionic radii taken from Shannon [S96]

	Sn-S	Sn-I	Sb-S	Sb-I
d, calculated	2.58 Å	3.16 Å	2.49 Å	3.05 Å
Σ_{Ionic}	3.02 Å	3.38 Å	2.60 Å	2.96 Å
$\Delta(d-\Sigma_{\text{Ionic}})$	-15 %	-7 %	-4 %	3 %

S2.2 DFT Functional Performance

The calculated lattice parameters of both the $Cmc2_1$ and *Cmcm* polymorphs, for the range of DFT functionals tested, are provided in Tables S2 to S5.

Note that while geometry relaxation with the PBEsol functional predicts lattice parameters in close agreement with experiment (approximately the same sum total of absolute errors in cell lengths as optB86b-vdW), we suspect this to be the result of a fortuitous cancellation of errors. This functional does not explicitly include any dispersion interactions, which are important in this material due to the atomic chain structure and lone-pair activity, and so optB86b-vdW was chosen as the DFT exchange-correlation functional for geometry relaxations in this investigation.

Indeed, this functional yields the closest agreement with experiment for the c/a lattice parameter ratio, a measure of the crystal unit cell shape and a common metric for the benchmarking of dispersion-corrected functional performance.[S97] Moreover, the ability of

Table S2 Calculated lattice parameters of the $Cmc2_1$ $Sn_2SbS_2I_3$ conventional orthorhombic unit cell, for the different DFT functionals tested. Errors given relative to the low temperature ($T = 173$ K) experimental values[S9,S11]

	c/a	**Δ(*c/a*)**	***a*** / Å	**Δ*a***	***b*** / Å	**Δ*b***	***c*** / Å	**Δ*c***	**Volume / Å^3**	**ΔVol.**
optB86b-vdW	3.85	−0.2%	4.29	0.9%	14.31	2.3%	16.51	0.8%	1013.8	4.1%
PBE-TS	3.87	0.5%	4.33	2.0%	15.15	8.3%	16.79	2.5%	1102.3	13.2%
HSE06	4.02	4.2%	4.27	0.6%	15.33	9.5%	17.16	4.8%	1124.4	15.5%
PBEsol	3.82	−0.9%	4.26	0.3%	14.24	1.8%	16.29	−0.6%	988.9	1.5%
**Experiment:**										
$T = 173$ K	3.85	—	4.25	—	13.99	—	16.38	—	973.9	—
$T = 293$ K	3.85	—	4.27	—	14.07	—	16.46	—	988.9	—

Table S3 Calculated lattice parameters of the $Cmcm$ $Sn_2SbS_2I_3$ conventional orthorhombic unit cell, for the different DFT functionals tested. Errors given relative to the low temperature ($T = 173$ K) experimental values[S9,S11]

	c/a	**Δ(*c/a*)**	***a*** / Å	**Δ*a***	***b*** / Å	**Δ*b***	***c*** / Å	**Δ*c***	**Volume / Å^3**	**ΔVol.**
optB86b-vdW	3.75	−2.8%	4.27	0.5%	14.02	0.2%	16.00	−2.3%	957.9	−2.8%
PBE-TS	3.69	−4.3%	4.33	1.9%	14.38	2.8%	15.97	−2.5%	994.9	2.2%
HSE06	3.71	−3.6%	4.29	0.9%	14.42	3.1%	15.92	−2.8%	984.6	1.1%
PBEsol	3.73	−3.3%	4.24	−0.3%	13.86	−0.9%	15.79	−3.6%	926.7	−6.2%
**Experiment:**										
$T = 173$ K	3.85	—	4.25	—	13.99	—	16.38	—	973.9	—
$T = 293$ K	3.85	—	4.27	—	14.07	—	16.46	—	988.9	—

this dispersion-corrected functional to incorporate van der Waals interactions in solids has been well demonstrated, yielding accurate predictions of lattice parameters in other lone-pair materials.[S19–S22]

S2.2.1 Room Temperature Comparison

Table S4 Calculated lattice parameters of the $Cmc2_1$ $Sn_2SbS_2I_3$ conventional orthorhombic unit cell, for the different DFT functionals tested. Errors given relative to the room temperature ($T = 293$ K) experimental values[S9,S11]

	c/a	**Δ(*c/a*)**	***a*** / Å	**Δ*a***	***b*** / Å	**Δ*b***	***c*** / Å	**Δ*c***	**Volume / Å^3**	**ΔVol.**
optB86b-vdW	3.85	−0.2%	4.29	0.4%	14.31	1.7%	16.51	0.3%	1013.8	2.5%
PBE-TS	3.87	0.5%	4.33	1.4%	15.15	7.6%	16.79	2.0%	1102.3	11.5%
HSE06	4.02	4.2%	4.27	0.1%	15.33	8.9%	17.16	4.3%	1124.4	13.7%
PBEsol	3.82	−0.9%	4.26	−0.2%	14.24	1.2%	16.29	−1.0%	988.9	0.0%
**Experiment:**										
$T = 293$ K	3.85	—	4.27	—	14.07	—	16.46	—	988.9	—
$T = 173$ K	3.85	—	4.25	—	13.99	—	16.38	—	973.9	—

Table S5 Calculated lattice parameters of the *Cmcm* $Sn_2SbS_2I_3$ conventional orthorhombic unit cell, for the different DFT functionals tested. Errors given relative to the room temperature ($T = 293\,K$) experimental values[S9,S11]

	c/a	Δ(*c/a*)	*a* / Å	Δ*a*	*b* / Å	Δ*b*	*c* / Å	Δ*c*	Volume / Å^3	ΔVol.
optB86b-vdW	3.75	−2.8%	4.27	0.0%	14.02	−0.4%	16.00	−2.8%	957.9	−3.1%
PBE-TS	3.69	−4.3%	4.33	1.4%	14.38	2.2%	15.97	−3.0%	994.9	0.6%
HSE06	3.71	−3.7%	4.29	0.4%	14.42	2.5%	15.92	−3.3%	984.6	−0.4%
PBEsol	3.73	−3.3%	4.24	−0.8%	13.86	−1.5%	15.79	−4.1%	926.7	−6.2%
**Experiment:**										
$T = 293\,K$	3.85	—	4.27	—	14.07	—	16.46	—	988.9	—
$T = 173\,K$	3.85	—	4.25	—	13.99	—	16.38	—	973.9	—

S3 Lone Pair Effect

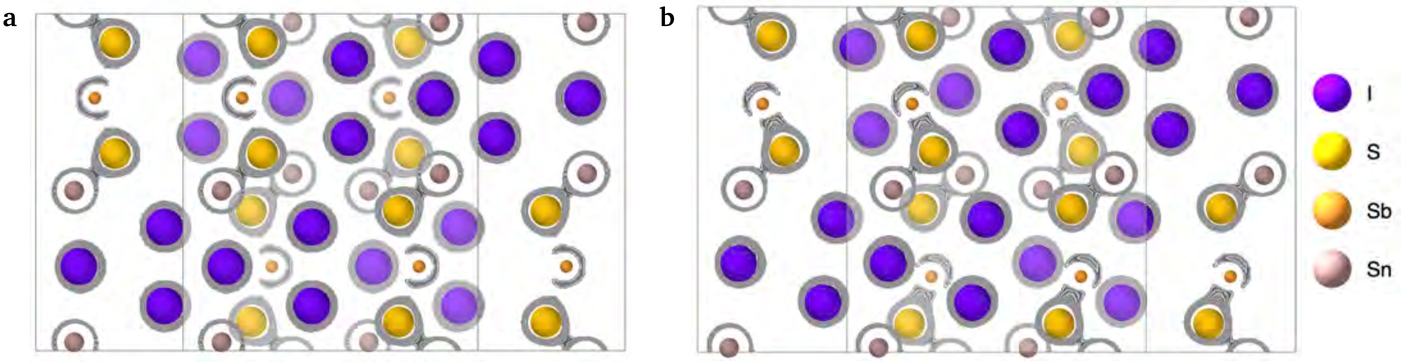

Fig. S6 Charge density contour plots projected onto the [110] orthorhombic cell plane, for the **(a)** *Cmcm* and **(b)** $Cmc2_1$ structural polymorphs of $Sn_2SbS_2I_3$. Note the $Cmc2_1$ polymorph has a slightly elongated cell length along the *c* direction, relative to *Cmcm*.

No distortion from spherical symmetry is apparent for the Sn(II) $5s^2$ electron pair, from the charge density alone. Using the Electron Localisation Function (ELF)[S98], however, a small degree of lone-pair localisation is witnessed.

This behaviour can be understood through analysis of the electronic density of states and simulated x-ray photoemission spectrum (XPS) in Figs. S13 and S20. We see that the Sb *s* states are essentially absent from the upper valence band, appearing instead as a localised peak at 10 eV below the VBM. In contrast, the Sn *s* states have a significant (anti-bonding) contribution at the VBM, and a partially-delocalised peak 6–8 eV below the VBM. This occurs as a result of the preferential alignment of Sn 5*s* with the anion *p* states (in the range -6 – $0\,eV$) due to a lower ionisation energy compared to Sb 5*s*.[S63] Consequently, the Sn $5s^2$ lone-pair has a stronger interaction with the anion *p* orbitals, resulting in charge delocalisation and minimal stereochemical activity. For Sb on the other hand, the localisation of $5s^2$ states in energy translates to spatial localisation of the lone-pair and dynamic stereochemical activity.

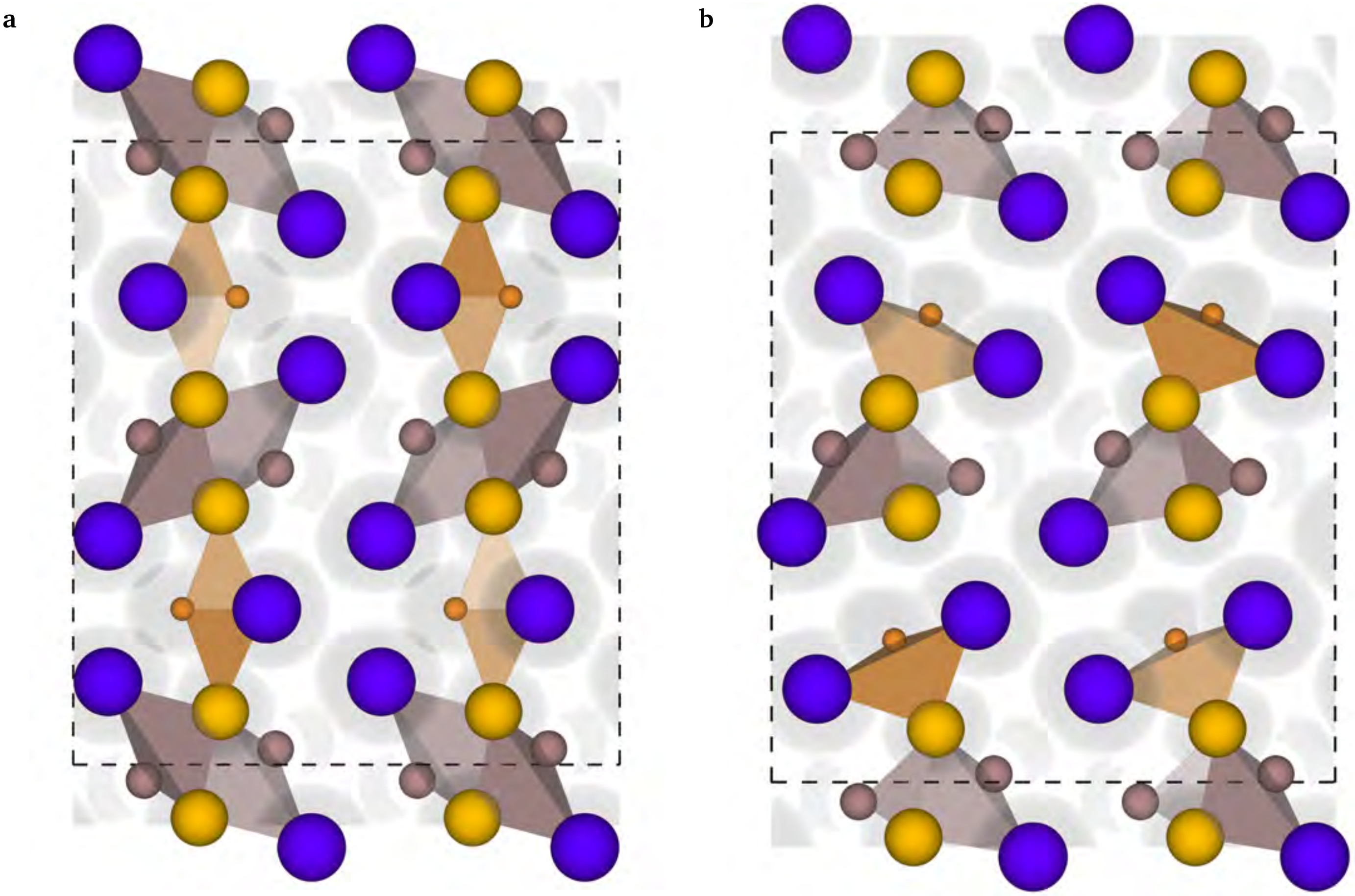

Fig. S7 Electron Localisation Function (ELF) contour plots projected onto the [110] orthorhombic cell plane, for the **(a)** *Cmcm* and **(b)** $Cmc2_1$ structural polymorphs of $Sn_2SbS_2I_3$. Note the slightly elongated cell length of the $Cmc2_1$ polymorph along the *c* direction, relative to *Cmcm*. Grayscale contours from ELF values of 0.5 to 1. Atoms sized according to their formal ionic radii.

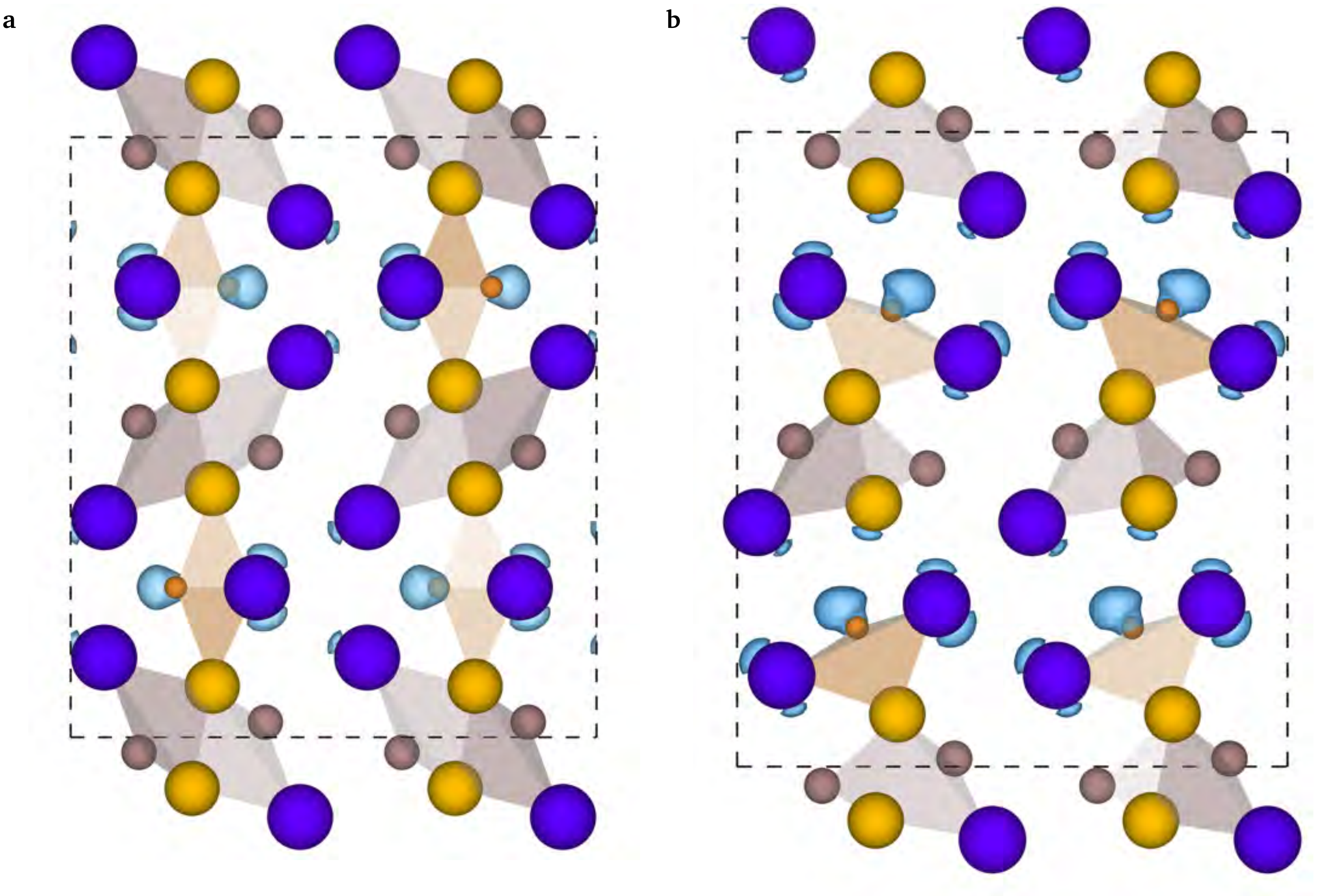

Fig. S8 Electron Localisation Function (ELF) isosurface plots projected onto the [110] orthorhombic cell plane, for the **(a)** *Cmcm* and **(b)** $Cmc2_1$ structural polymorphs of $Sn_2SbS_2I_3$. Note the $Cmc2_1$ polymorph has a slightly elongated cell length along the c direction, relative to *Cmcm*. Isovalue set to 0.9. Atoms sized according to their formal ionic radii.

S4 Dielectric Response

Table S6 Born effective charge tensors (Z^*_{ii}; $|e|$) for each *symmetry-inequivalent* atomic species in **(left)** $Cmc2_1$ and **(right)** *Cmcm* $Sn_2SbS_2I_3$, calculated using the optB86b-vdW DFT functional.[a] Comparison given to their formal oxidation states

Species ($Cmc2_1$)	Ox. State	Z^*_{xx}	Z^*_{yy}	Z^*_{zz}
Sn_a	+2	+3.93	+2.74	+2.85
Sn_b	+2	+4.21	+3.59	+2.67
Sb	+2	+6.17	+2.84	+6.08
S_a	−2	−2.12	−2.42	−4.37
S_b	−2	−3.26	−2.51	−2.63
I_a	−1	−2.70	−0.76	−1.16
I_b	−1	−3.31	−1.46	−0.48
I_c	−1	−2.92	−2.01	−2.09

Species (*Cmcm*)	Ox. State	Z^*_{xx}	Z^*_{yy}	Z^*_{zz}
Sn	+2	+4.00	+3.00	+3.41
—	—	—	—	—
Sb	+2	+6.82	+3.55	+9.08
S	−2	−2.88	−2.44	−4.40
—	—	—	—	—
I_a	−1	−2.97	−0.93	−1.31
I_b	−1	−3.06	−1.87	−2.89
—	—	—	—	—

a The Born effective charge is a measure of the relationship between lattice polarisation and atomic displacement, and is greater for the *Cmcm* phase due to structural instability.[S47,S49]

Both the Born effective charges and dielectric response show strong anisotropy, with greater magnitudes along the *a* and *c* directions (*i.e.* along the atomic chains and where the interchain contact/bonding is greatest), due to the presence of stereochemically-active lone-pairs and anisotropic crystal structure.

Table S7 Dielectric tensors (ε^*_{ii}) for $Cmc2_1$ $Sn_2SbS_2I_3$, calculated using the optB86b-vdW DFT functional

Dielectric Response	ε_{xx}	ε_{yy}	ε_{zz}
Ionic, (ε_{Ionic})	41.24	9.38	13.92
Electronic, (ε_∞)	10.03	8.85	8.44
Total, (ε_0)	51.27	18.23	22.36

S5 Band Alignment

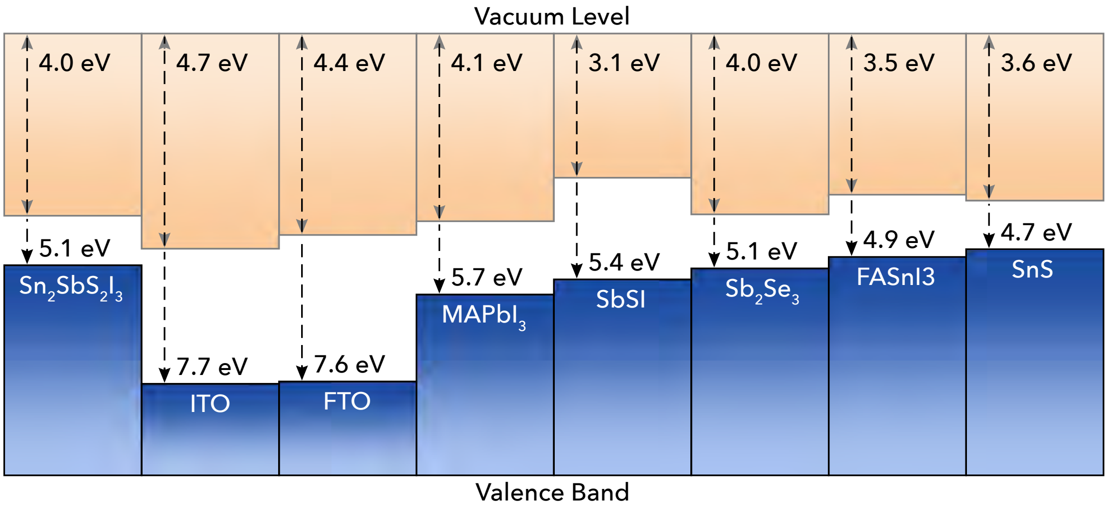

Fig. S9 Calculated band alignment of $Sn_2SbS_2I_3$, showing the ionisation potential and electron affinity, alongside those of indium tin oxide (ITO),[S99] fluorine-doped tin oxide (FTO),[S100] $MAPbI_3$,[S101] SbSI,[S30] Sb_2Se_3,[S35] $FaSnI_3$[S73] and SnS[S102] for comparison.

The calculated electronic band alignment of $Sn_2SbS_2I_3$ agrees well with that measured by Nie *et al.*[S4], who determined an electron affinity of 4.17 eV and ionisation potential of 5.58 eV using an FTO/$Sn_2SbS_2I_3$ device. One possible cause of discrepancy between theory and experiment, in this case, is that surface and interfacial dipoles can result in shifts of electron band positions, relative to the 'natural' band position calculated by theory.

S6 Additional Electronic and Optical Analysis

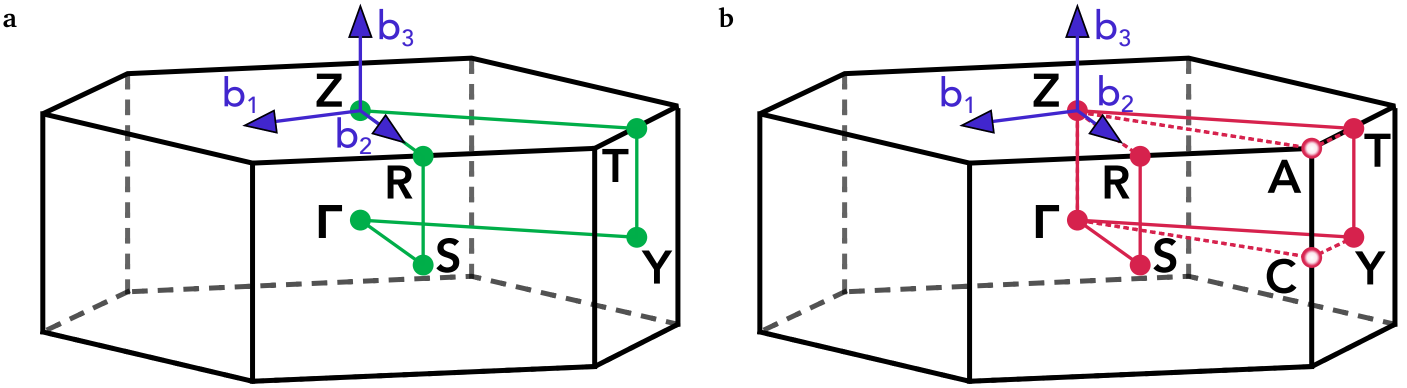

Fig. S10 First Brillouin Zone (BZ) of a C-centred orthorhombic lattice,[S103] to which space groups *Cmcm* and $Cmc2_1$ belong. The high-symmetry BZ points and band path included in the **(a)** phonon dispersions (Fig. 2) and **(b)** electronic band structure (Fig. 5a) are shown in colour. The additional **k**-points and reciprocal space path included in the expanded band structures in Fig. S11 are depicted by hollow circles and dotted lines respectively. Vectors $\mathbf{b}_{1,2,3}$ denote the cell vectors of the Wigner-Seitz primitive cell. Note that labels **C** and **A** are also equivalent to **Σ** and **E** respectively.

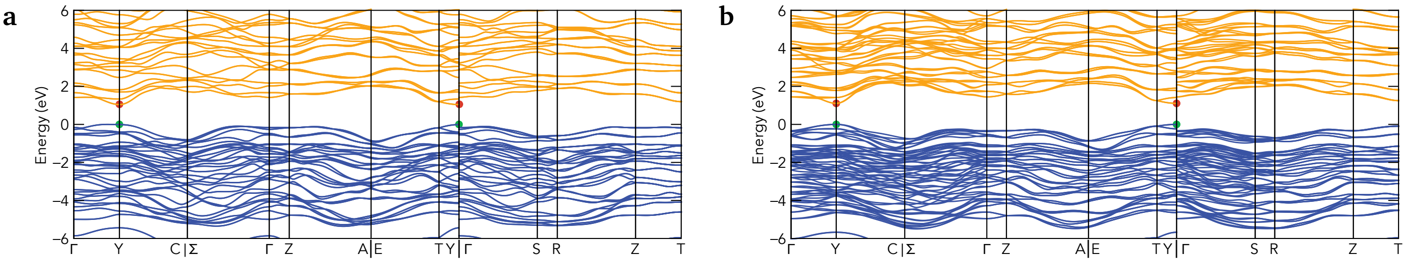

Fig. S11 Electronic band structures of $Sn_2SbS_2I_3$ in the **(a)** *Cmcm* and **(b)** $Cmc2_1$ crystal structures. Valence band in blue, conduction band in orange and band extrema highlighted. VBM set to 0 eV. **k**-point path generated with `SeeKpath`[S104] and band structure diagrams produced with `Sumo`.[S59] Brillouin zone path shown in Fig. S10b.

The *Cmcm* polymorph of $Sn_2SbS_2I_3$ is found to have a pseudo-direct bandgap, with an indirect fundamental gap of 1.02 eV (VBM along the $\boldsymbol{Y} \rightarrow \boldsymbol{\Gamma}$ high-symmetry k-point path, near $\boldsymbol{Y}$; CBM at $\boldsymbol{Y}$) and a direct gap of 1.03 eV at $\boldsymbol{Y}$. For the $Cmc2_1$ polymorph, we calculate a direct fundamental gap of 1.08 eV at $\boldsymbol{Y}$.
Using non-parabolic fitting of the band-edges, the light and heavy carrier effective masses (for $Cmc2_1$ $Sn_2SbS_2I_3$) are calculated as $m_e^* = 0.19\,\mathrm{m_0}$ ($\boldsymbol{Y} \rightarrow \boldsymbol{C}$) & $0.81\,\mathrm{m_0}$ ($\boldsymbol{Y} \rightarrow \boldsymbol{T}$), and $m_h^* = 0.49\,\mathrm{m_0}$ ($\boldsymbol{Y} \rightarrow \boldsymbol{C}$) & $1.37\,\mathrm{m_0}$ ($\boldsymbol{Y} \rightarrow \boldsymbol{T}$) for electrons and holes respectively. For *Cmcm* $Sn_2SbS_2I_3$; $m_e^* = 0.21\,\mathrm{m_0}$ ($\boldsymbol{Y} \rightarrow \boldsymbol{C}$) & $0.81\,\mathrm{m_0}$ ($\boldsymbol{Y} \rightarrow \boldsymbol{T}$), and $m_h^* = 1.61\,\mathrm{m_0}$ ($\boldsymbol{Y} \rightarrow \boldsymbol{C}$) & $2.22\,\mathrm{m_0}$ ($\boldsymbol{Y} \rightarrow \boldsymbol{T}$). Effective masses obtained using harmonic fitting do not deviate significantly ($\Delta m^* < 0.05\,\mathrm{m_0}$) from these values, save for $m^*_{h,Cmcm}$ (for which $\Delta m_h^* \sim 0.1\,\mathrm{m_0}$).

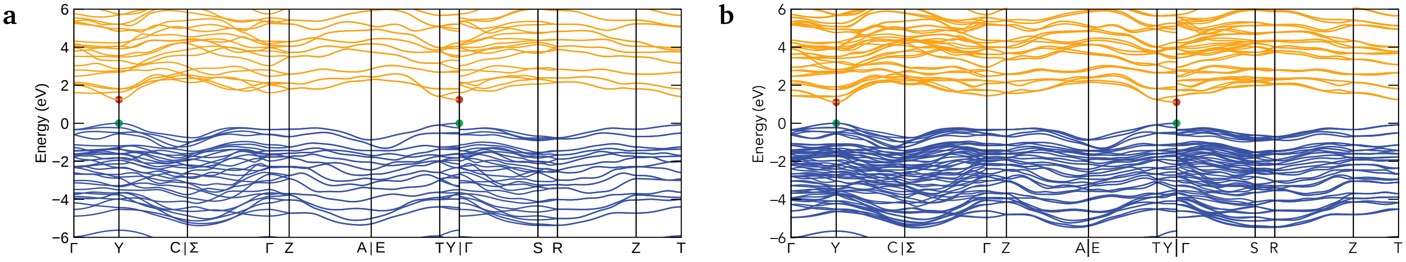

Fig. S12 Electronic band structures of $Cmc2_1$ $Sn_2SbS_2I_3$ **(a)** excluding and **(b)** including spin-orbit coupling (SOC) effects. Valence band in blue, conduction band in orange and *direct gap* band extrema highlighted. VBM set to 0 eV. Generated with `Sumo`.[S59]

The calculated fundamental electronic bandgap reduces from 1.22 eV (HSE06, Fig. S12a) to 1.08 eV (HSE06+SOC, Fig. S12b) upon inclusion of spin-orbit coupling effects.

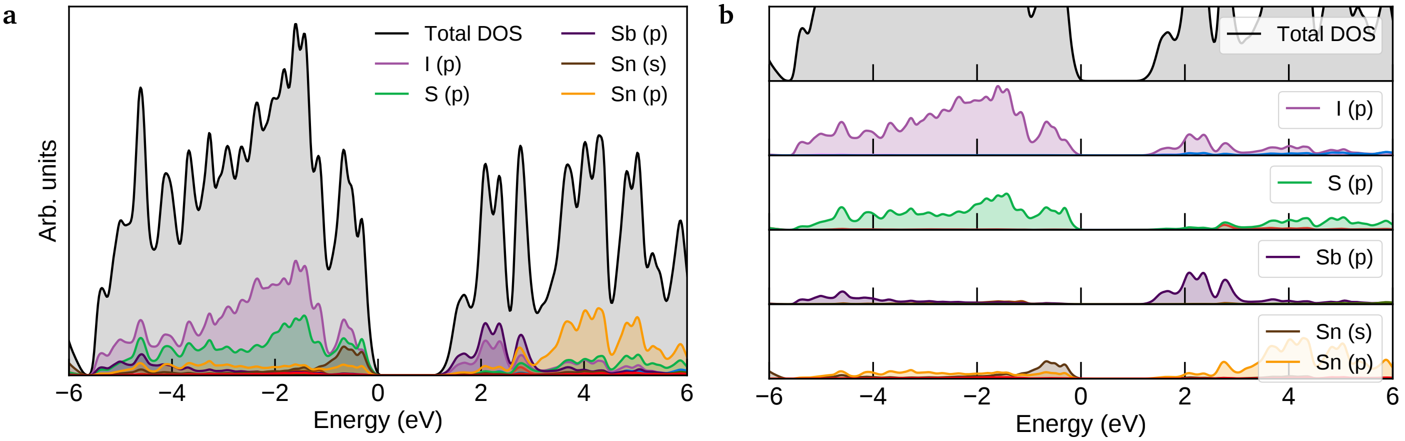

Fig. S13 Orbital-projected electronic density of states of $Cmc2_1$ $Sn_2SbS_2I_3$ with summed (**a**) and separated (**b**) contributions. VBM set to 0 eV. Generated with `Sumo`.[S59]

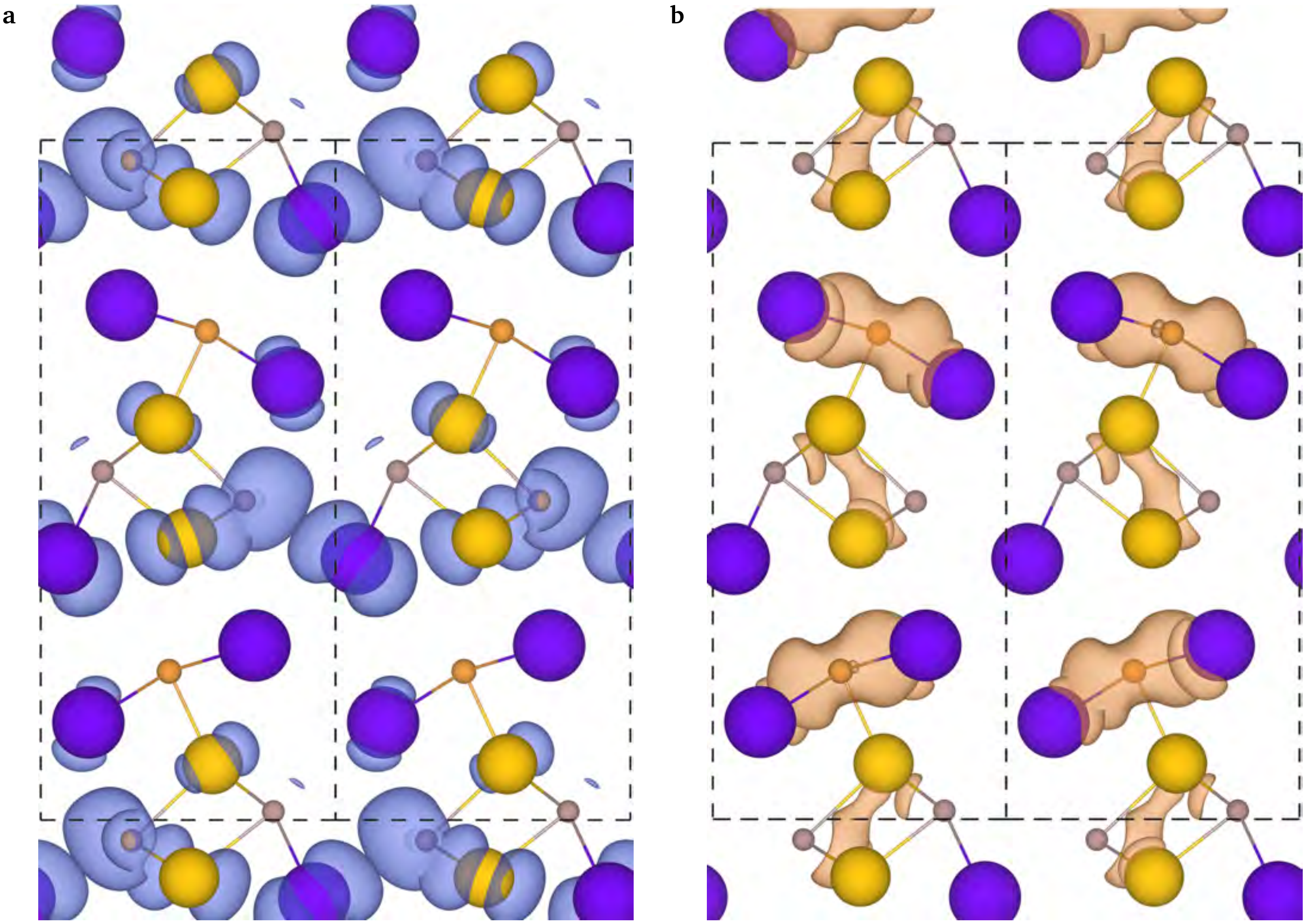

Fig. S14 Charge density isosurfaces of the **(a)** valence band maximum (VBM) and **(b)** conduction band minimum (CBM) states of $Cmc2_1$ $Sn_2SbS_2I_3$, both of which are located at the $\boldsymbol{Y}$ high-symmetry k-point. Isovalue set to 1×10^{-5}.

To elucidate the optical behaviour of $Sn_2SbS_2I_3$ in the $Cmc2_1$ polymorphic ground-state, it is instructive to first consider the behaviour of the metastable *Cmcm* structure, neglecting SOC effects initially for demonstration purposes. Fig. S15 shows a breakdown of the contributing factors to the overall optical absorption of the *Cmcm* polymorph. The overall absorption spectrum is essentially derived from the convolution of the joint density of states (JDOS) — the summation of all possible vertical electronic transitions at a given energy — with the transition probability, via Fermi's Golden Rule.[S105]

In the centrosymmetric *Cmcm* structure, the even (*gerade*) parity of both the VBM and CBM wavefunctions at $\boldsymbol{Y}$ (Fig. S11) about the crystal inversion centre results in a formally symmetry-forbidden transition at the direct (optical) gap, as witnessed in Fig. S15a,b,d. This is a result of the fact that the electric dipole operator $\vec{\mu}$ has odd parity, so the equal parities of the initial and final electronic states ($|i\rangle$ and $|f\rangle$) result in an overall odd function within the transition dipole matrix element $\langle i|\vec{\mu}|f\rangle$, which then becomes zero when integrated over space — thus yielding zero overall transition probability between these electronic states.[S105]

Away from $\boldsymbol{Y}$, and for the VBM+1 and CBM+1 wavefunctions, this is no longer the case, and so the transition probabilities (Fig. S15a,b) and optical absorption (Fig. S15d) begin to rise rapidly around 0.4 eV above the direct gap.

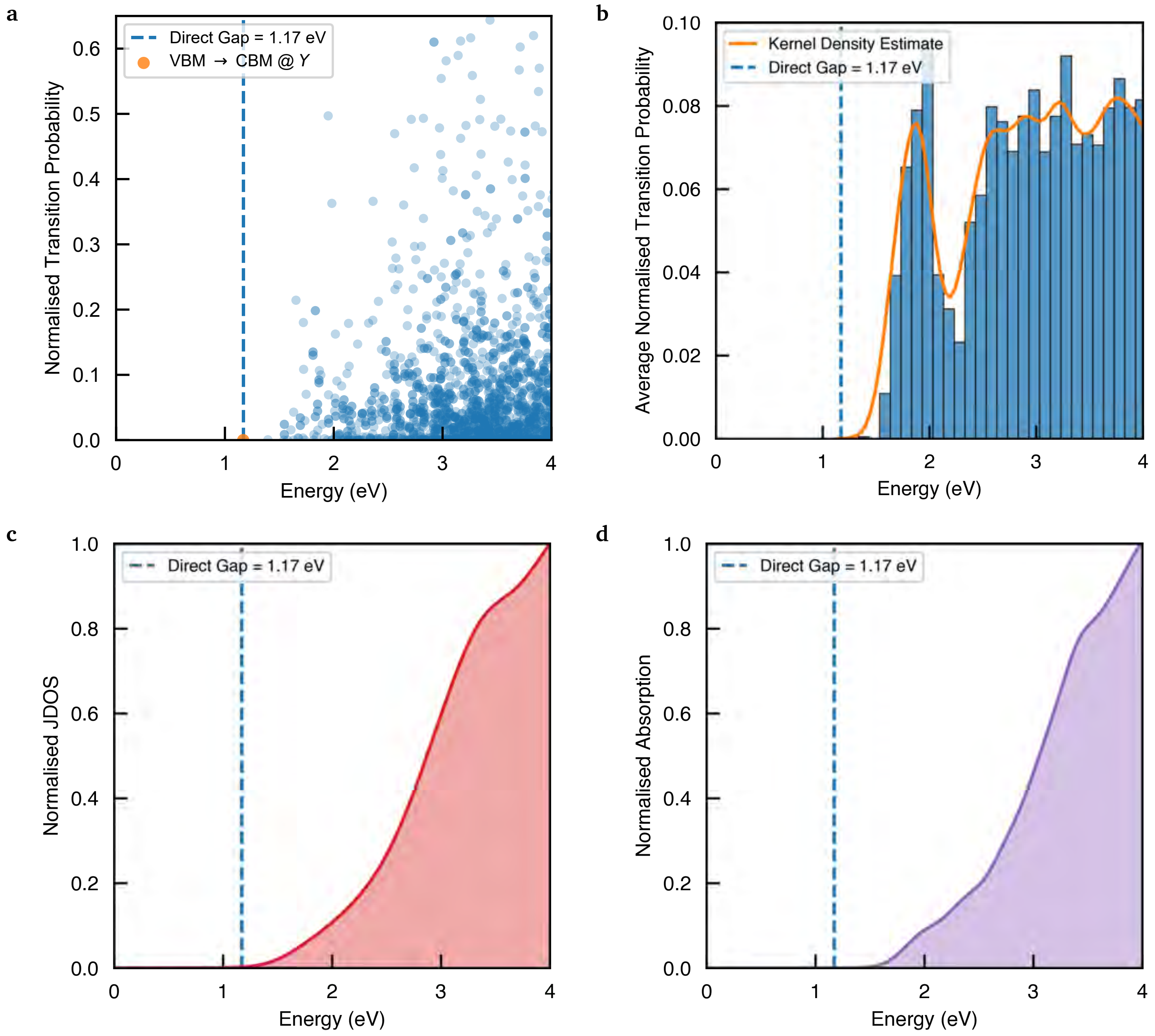

Fig. S15 Contributing factors to the optical absorption behaviour in the metastable *Cmcm* polymorph of $Sn_2SbS_2I_3$, neglecting spin-orbit coupling (SOC) effects for demonstration purposes. **a.** Transition probabilities for each vertical electronic transition in the optical absorption calculation. Given as a function of energy and normalised to the maximum value within the $0-4\,\mathrm{eV}$ range. **b.** Average normalised transition probabilities in energy regions of width $0.1\,\mathrm{eV}$. Then Kernel Density Estimate (KDE) gives a continuous probability density function via Gaussian smoothing of the data. **c.** Joint Density of States (JDOS) as a function of energy, normalised to the value at $E=4\,\mathrm{eV}$. **d.** Optical absorption spectrum, normalised to the value at $E=4\,\mathrm{eV}$.

The relatively low density of transitions (*i.e.* scatter points) just above the bandgap energy in Fig. S15a is due to the low density of states in the immediate vicinity ($\sim 0.3\,\mathrm{eV}$) of the VBM/CBM (Fig. S11a), in combination with the finite sampling of $\boldsymbol{k}$-space in the optical absorption calculation. Convergence of the dielectric function (*i.e.* high-frequency dielectric constant and optical absorption) with respect to $\boldsymbol{k}$-point sampling was carefully checked and confirmed, so this does not affect the predicted absorption behaviour, with the transition probabilities following the trends shown in Fig. S15a and Fig. S15b.

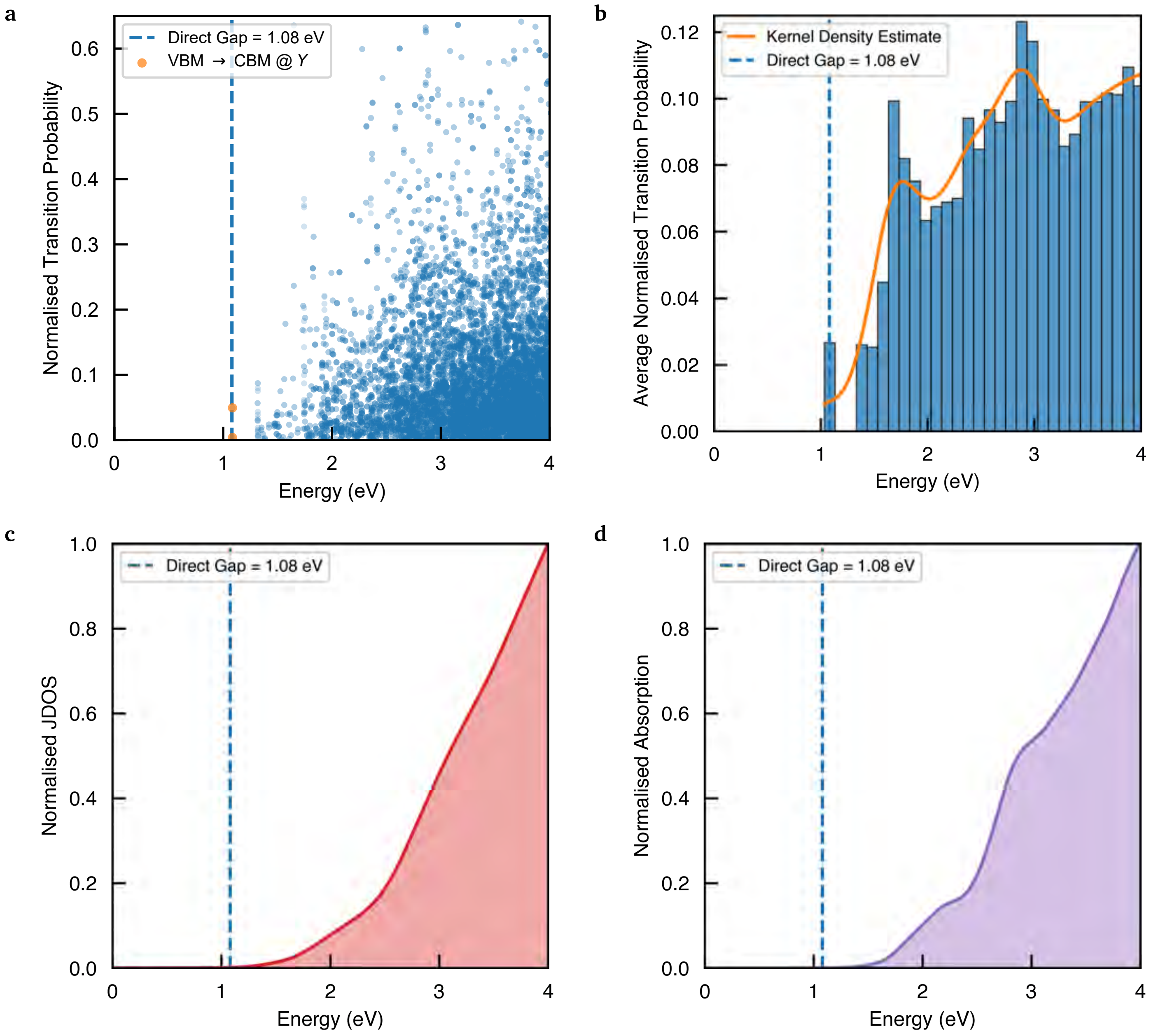

Fig. S16 Contributing factors to the optical absorption behaviour in the ground-state $Cmc2_1$ polymorph of $Sn_2SbS_2I_3$, including spin-orbit coupling (SOC) effects. **a.** Transition probabilities for each vertical electronic transition in the optical absorption calculation. Given as a function of energy and normalised to the maximum value within the $0-4$ eV range. **b.** Average normalised transition probabilities in energy regions of width 0.1 eV. Then Kernel Density Estimate (KDE) gives a continuous probability density function via Gaussian smoothing of the data. **c.** Joint Density of States (JDOS) as a function of energy, normalised to the value at $E = 4$ eV. **d.** Optical absorption spectrum, normalised to the value at $E = 4$ eV.

Turning now to the non-centrosymmetric $Cmc2_1$ ground-state phase, we see that this symmetry selection rule is broken by both the shift in Sb positions (removing the inversion symmetry) and spin-orbit splitting. Consequently, the direct gap transition at ***Y*** is no longer formally forbidden, as demonstrated by the finite transition probability at $E = 1.08$ eV in Fig. S16a,b. However, the effects of inversion symmetry-breaking and spin-orbit splitting represent a relatively minor perturbation to the electronic structure (Figs. S11 and S12), and so the transition dipole matrix element remains weak near the fundamental gap, due to symmetry restraints in combination with the spatial separation of the VBM and CBM states (Fig. S14).

The second conduction band (*i.e.* CBM+1), with energy $E = E_{CBM} + 0.3$ eV, is of odd (*ungerade*) parity in the *Cmcm* structure (parity is undefined in the non-centrosymmetric $Cmc2_1$ structure). The reduced symmetry restriction leads to larger transition probabilities from the VBM to this electronic state, in both polymorphs (Fig. S15a,b and Fig. S16a,b), at $E \geq 1.4$ eV. This trend in the optical transition dipole matrix (TDM), in combination with a particularly low joint density of states above the bandgap energy (Fig. S16), produces a weak but non-zero absorption at the direct bandgap $E = 1.08$ eV, with a sharp increase around $1.4-1.6$ eV.

Plotting the integral-normalised joint density of states (JDOS) alongside the integral-normalised optical absorption spectrum as in Fig. S17, we obtain a further demonstration of the weak TDM for the VBM $\rightarrow$ CBM transition. The normalised JDOS is significantly reduced relative to the optical absorption at energies just above the fundamental bandgap, leading to an 'optical band gap' greater than the fundamental direct gap.

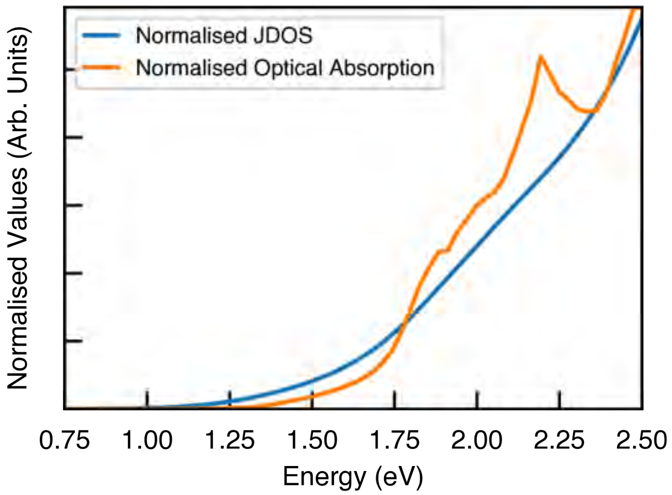

Fig. S17 Joint Density of States (JDOS) plotted alongside the optical absorption, where the quantities have been normalised by equating their integral sum from 0 to 5 eV. JDOS calculated using `VASPkit`[S106].

We propose this optical behaviour as a likely cause of the apparent mismatch between the calculated fundamental band gap ($E_g = 1.08\,\text{eV}$) and that measured by optical spectroscopy ($E_{\text{Opt, Exp}} = 1.41\,\text{eV}$). While the slow onset of absorption renders the unambiguous determination of an optical gap difficult, Tauc-plot fitting of the calculated absorption within the 1–1.8 eV range gives a best linear fit (with $R^2 = 0.93$) for an optical gap in the range 1.4–1.5 eV — as shown in Fig. S18. Indeed, this plot closely resembles that measured by Nie *et al.* [S4], who also reported a large Urbach energy of 464 meV — which may in part be an artefact of the slow absorption onset.

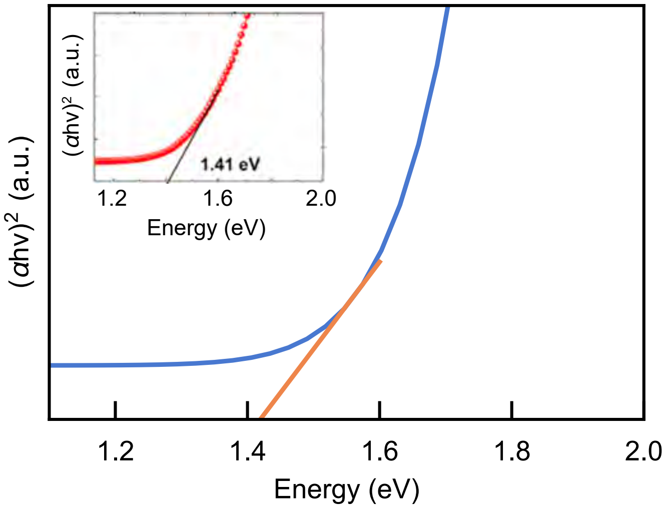

Fig. S18 Tauc plot of the optical absorption, assuming a direct allowed band gap transition ($r = 1/2$), compared to that measured by Nie *et al.* [S4] **(inset)**. Inset figure reproduced with permission from Ref. S4.

It should be noted that the hybrid DFT functional, HSE06, has been observed to underestimate electronic bandgaps in halide perovskites,[S64] and so the possiblity of similar behaviour in this material class cannot be ruled out.

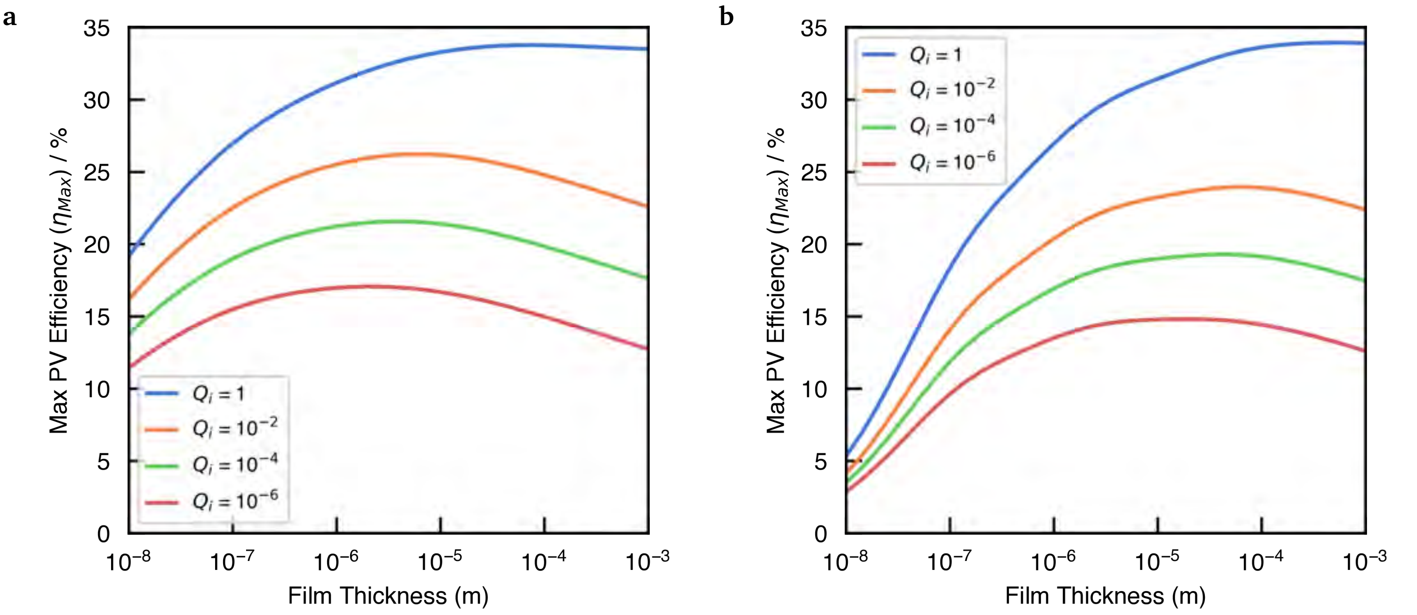

Fig. S19 Maximum PV efficiency (η_{max}) as a function of film thickness for a range of internal quantum luminescence efficiencies (Q_i), using the Blank *et al.*[S61] metric and assuming a **(a)** Lambertian scattering and **(b)** flat front surface.

These results indicate that the use of a Lambertian scattering front surface with an absorber thickness of $t \sim 2\,\mu$m would allow for maximal photovoltaic efficiencies with this material. For a flat front surface, absorber thicknesses of $t > 4\,\mu$m are predicted to be optimal for achieving high efficiencies. We note that Nie *et al.*[S4] used an active layer thickness of approximately $2\,\mu$m, and so we propose greater thicknesses may yield higher efficiencies (assuming a flat front surface).

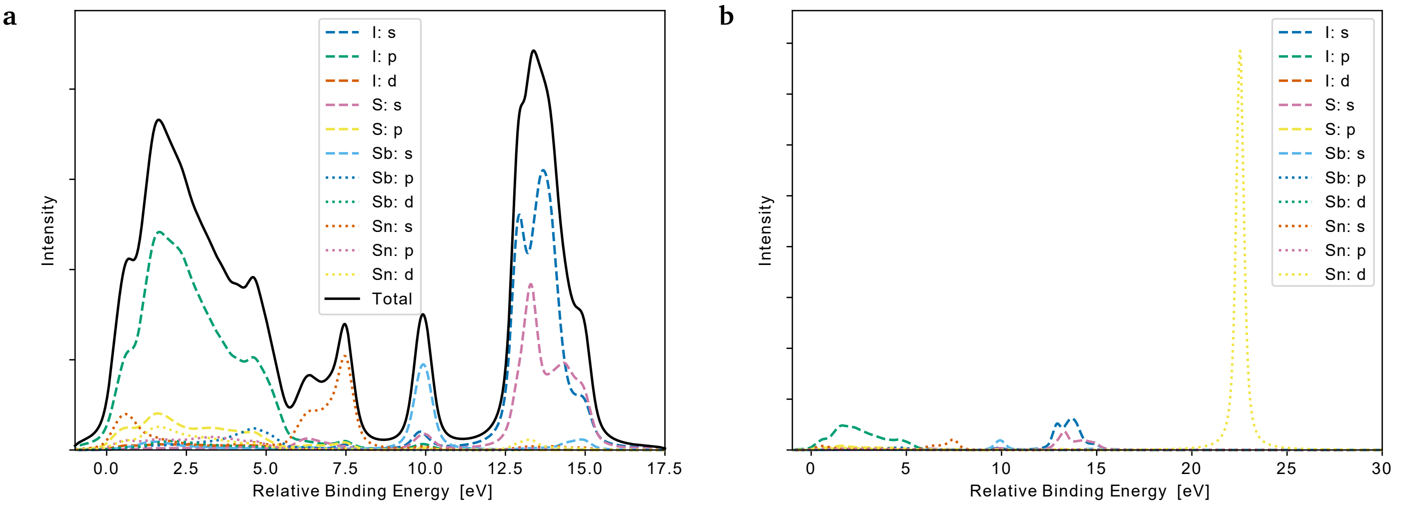

Fig. S20 Simulated x-ray photoelectron excitation spectrum (XPS) of $Sn_2SbS_2I_3$, showing the total and orbital-decomposed contributions down to 17.5 eV below the VBM (**a**) and orbital-decomposed down to 30 eV below the VBM (**b**). The large Sn d peak at occurs at 22.5 eV below the VBM, (27.5 eV below the vacuum level, using the calculated value for the ionisation potential $I = 5.06$ eV. X-axis is the electron binding energy in eV, and the VBM is set to 0 eV. Gaussian and Lorentzian broadening each of width 0.3 eV applied to match typical experimental values. Photoionisation cross-section weightings correspond to Al Kα radiation. Generated with `Galore`.[S77]

S6.1 Bonding Analysis via the Crystal Orbital Hamiltonian Population (COHP)[S71]

Crystal Orbital Hamilton Population analysis (COHP) involves partitioning the band-structure energy of a material into a sum of pairwise atomic orbital interactions.[S70,S71] It is defined as:

$$\mathrm{COHP}_{\mu\nu}(E) = H_{\mu\nu}(E)P_{\mu\nu}(E) \tag{S2}$$

$$H_{\mu\nu} = \langle \phi_\mu | \hat{H} | \phi_\nu \rangle \quad \text{(S3)}$$

$$P_{\mu\nu} = \sum_i f_i c^*_{\mu i} c_{\nu i} \delta(\varepsilon - \varepsilon_i) \quad \text{(S4)}$$

In essence, COHP analysis indicates bonding, nonbonding, and antibonding energy regions in the electronic density of states, providing a powerful tool for the inspection of chemical bonding behaviour in materials. As such, the integrated Crystal Orbital Hamilton Population (ICOHP) for a given pair of atoms or orbitals (i.e. the energy contribution to the band-structure energy) can be viewed as a *measure* of the total energetic contribution of the (anti-)bonding interaction of that pair to the system energy.

$$\text{ICOHP}_{\mu\nu}(E) = \int_{-\infty}^{\varepsilon_f} \text{COHP}_{\mu\nu}(E)\, dE \quad \text{(S5)}$$

A negative value for the (I)COHP indicates an (overall) energy lowering orbital interaction (i.e. bonding), while a positive value indicates anti-bonding type interactions.

Orbital-projected COHP analysis was performed for all possible cation-anion combinations of Sn $4d$, $5s$ and $5p$, Sb $5s$ and $5p$, S $3s$ and $3p$, and I $5s$ and $5p$. Those with significant contributions near the band edges are plotted below. Note the differences in axis limits for different orbital interactions.

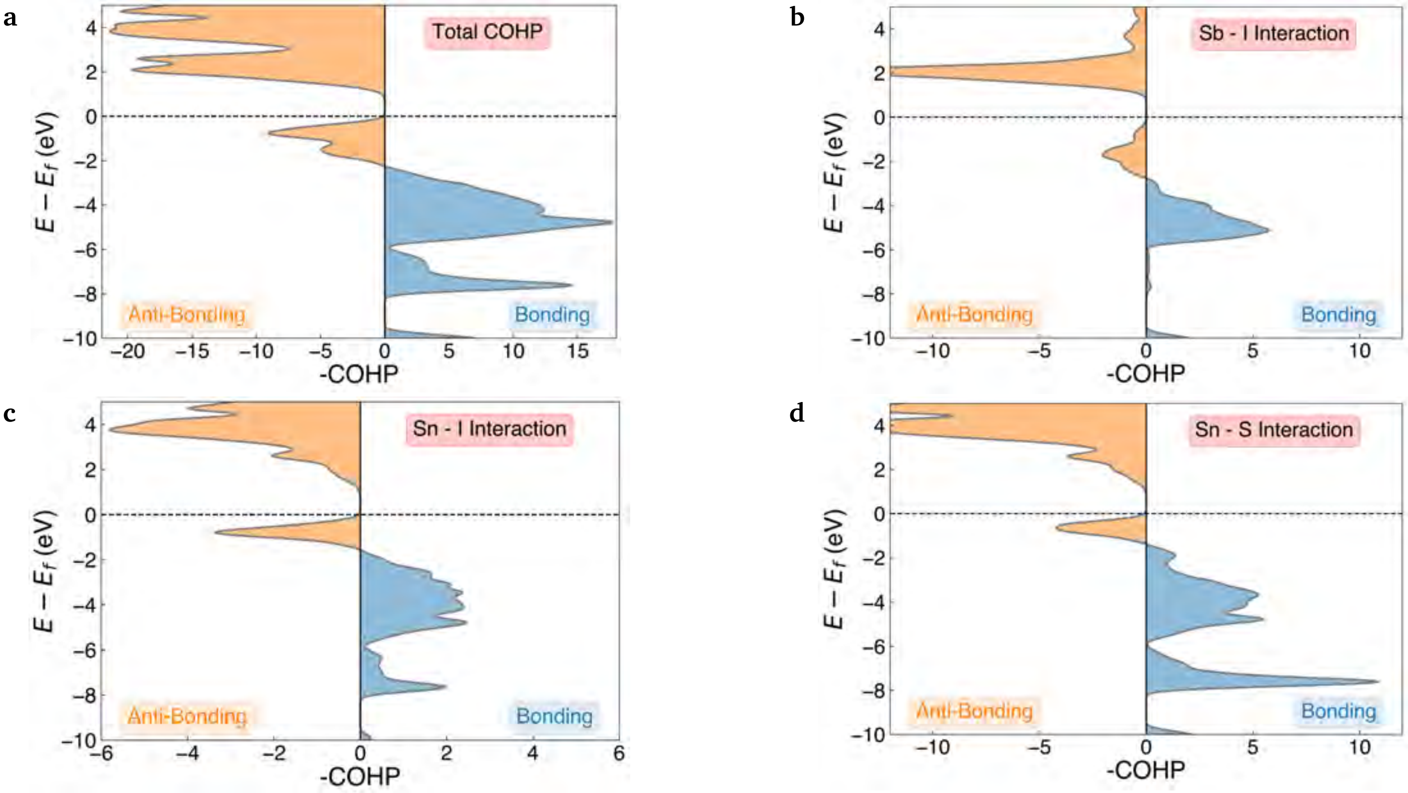

Fig. S21 COHP(E) analysis of the **(a)** total, **(b)** Sb - I, **(c)** Sn - I and **(d)** Sn - S interactions in $Sn_2SbS_2I_3$.

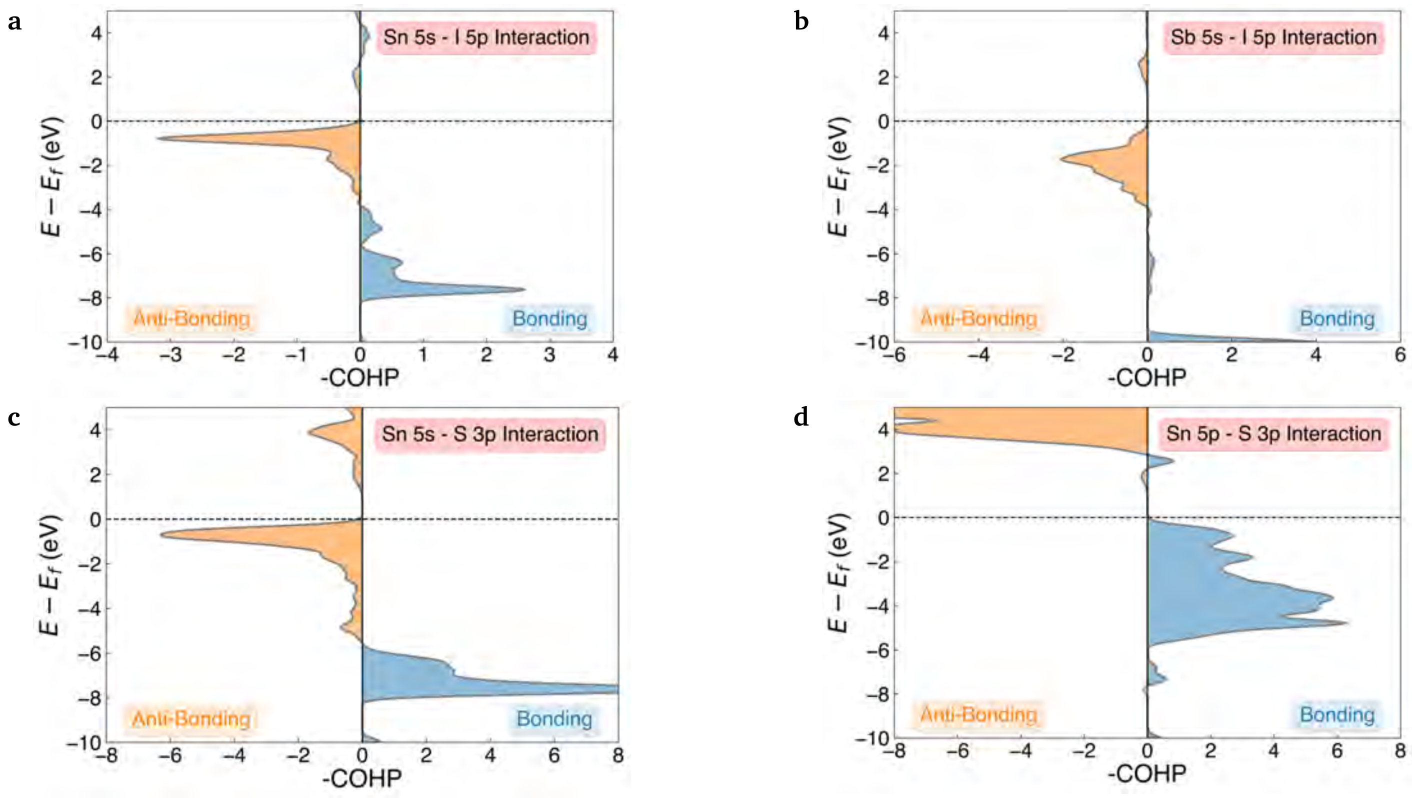

Fig. S22 COHP(E) orbital-decomposed analysis of the **(a)** Sn $5s$ - I $5p$, **(b)** Sb $5s$ - I $5p$, **(c)** Sn $5s$ - S $3p$ and **(d)** Sn $5p$ - S $3p$ interactions in $Sn_2SbS_2I_3$.

S7 Nudged Elastic Band (NEB) Calculations

To calculate the energetic barrier to switching of the polarisation direction, the Nudged Elastic Band (NEB)[S50] method was employed to map out the potential energy surface (PES) along the minimum-energy path between equivalent $Cmc2_1$ configurations (Figs. 3 and S23). The `optB86b-vdW` dispersion-corrected DFT functional was used for the NEB geometry relaxations, and the energies of the final structures were then calculated with the Random Phase Approximation (RPA) to the correlation energy, using electronic wavefunctions calculated with the HSE06[S23] screened hybrid DFT functional.[S24,S25]

Employing the standard implementation of the NEB method, where a linear interpolation between equivalent $Cmc2_1$ configurations is used to generate the initial structures, we find the non-polar $Cmcm$ phase to correspond to the transition state between polar $Cmc2_1$ states, with no local stability around this saddle point on the PES. To confirm the lack of a local energy minimum about the $Cmcm$ structure, on this structural path, this calculation was repeated with a dense sampling of the PES (Fig. S24) — using the energies predicted by the `optB86b-vdW` functional in this case (RPA energies are computationally intractable for this number of structures).

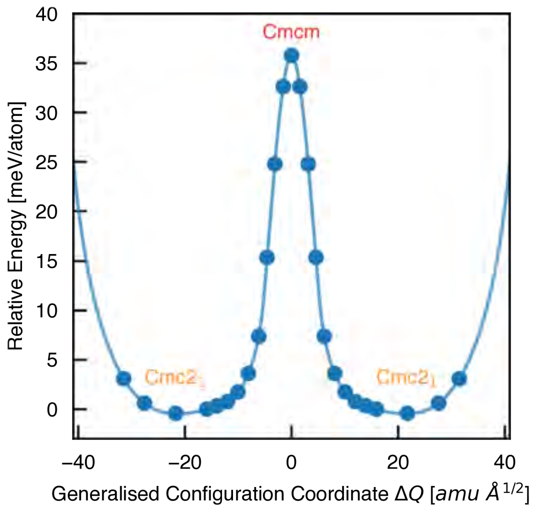

Fig. S23 Potential energy surface along the configurational path between equivalent $Cmc2_1$ configurations of $Sn_2SbS_2I_3$ (corresponding to the curved arrows in Fig. 1), calculated using the Nudged Elastic Band (NEB) method.[S50] Filled circles represent calculated data points and the solid line is a spline fit. X axis given in units of mass-weighted displacement.

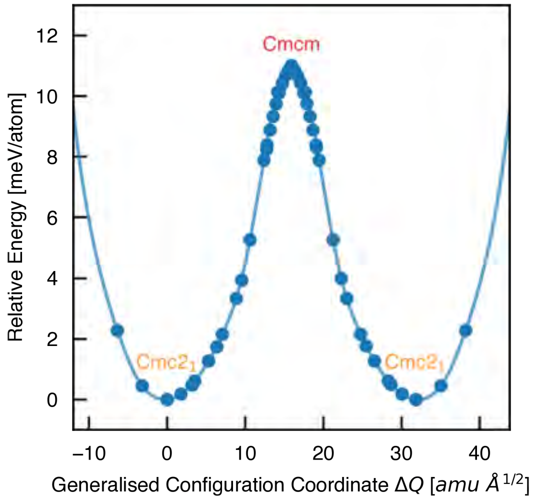

Fig. S24 Sombrero potential energy surface along the configurational path between equivalent $Cmc2_1$ configurations of $Sn_2SbS_2I_3$ (corresponding to the curved arrows in Fig. 1), calculated using the `optB86b-vdW` DFT functional within the Nudged Elastic Band (NEB) method.[S50] In this case, the formation enthalpy difference is calculated as $\Delta E = 11.1\,\mathrm{meV/atom}$, due to the use of the `optB86b-vdW` DFT functional.

To confirm that the *Cmcm* phase does indeed represent the saddle point structure along the *minimum energy* path between $Cmc2_1$ configurations, the Distortion Symmetry Method developed by Munro *et al.*[S107] was employed. This method enumerates the possible lower-symmetry pathways to ferroelectric switching, which can then be used as starting-points for NEB calculations, to determine whether a lower-symmetry, lower-energy-barrier pathway is present. In our case, we find that no lower-energy pathway was present, and thus that the energetic barrier to ferroelectric switching corresponds to the relative energy of the *Cmcm* and $Cmc2_1$ polymorphs; $\Delta E = 35.8\,\mathrm{meV/atom}$.

S8 *Ab initio* Molecular Dynamics (AIMD)

To further probe the polarisation dynamics and stability in $Sn_2SbS_2I_3$, several Molecular Dynamics (MD) simulations were carried out at room temperature ($T = 300\,\mathrm{K}$) and at 227 °C ($T = 500\,\mathrm{K}$). In the non-polar *Cmcm* arrangement, Sb has two equivalent nearest-neighbour S atoms, in the atomic chains above and below in the c direction. In switching to the polar $Cmc2_1$ configuration, Sb moves along the c axis in the direction of polarisation, decreasing the distance to one S neighbour (within the a-direction stoichiometric chain) and

increasing the distance to the other S neighbour (in the opposing chain, Fig. S2) – as shown by the radial distribution functions and probability densities in Fig. S25. This allows the polarisation dynamics of $Sn_2SbS_2I_3$ during MD simulations to be visualised through the Sb–S bond lengths, with a switch of the polarisation direction (and hence Sb position) corresponding to swapping of the short and long Sb–S bonds.

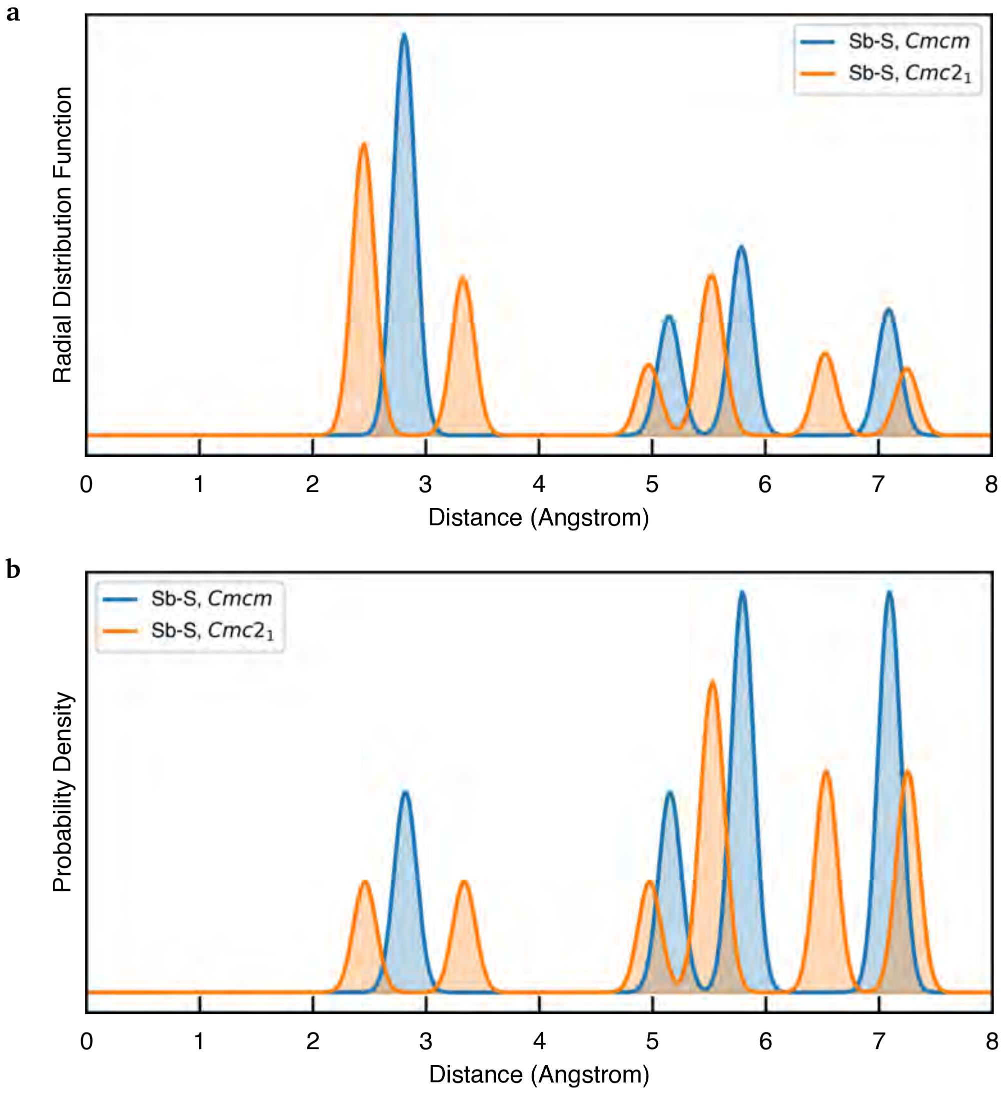

Fig. S25 Smeared radial distribution function **(a)** and probability density **(b)** between Sb and S ions for both structural polymorphs of $Sn_2SbS_2I_3$, relaxed with the PBE-TS DFT functional. Probability densities given by $4\pi r^2 g(r)$, where $g(r)$ is the radial distribution function.

Fig. S26 and Fig. S27 show the results of MD simulations performed at room temperature ($T = 300\,\mathrm{K}$).

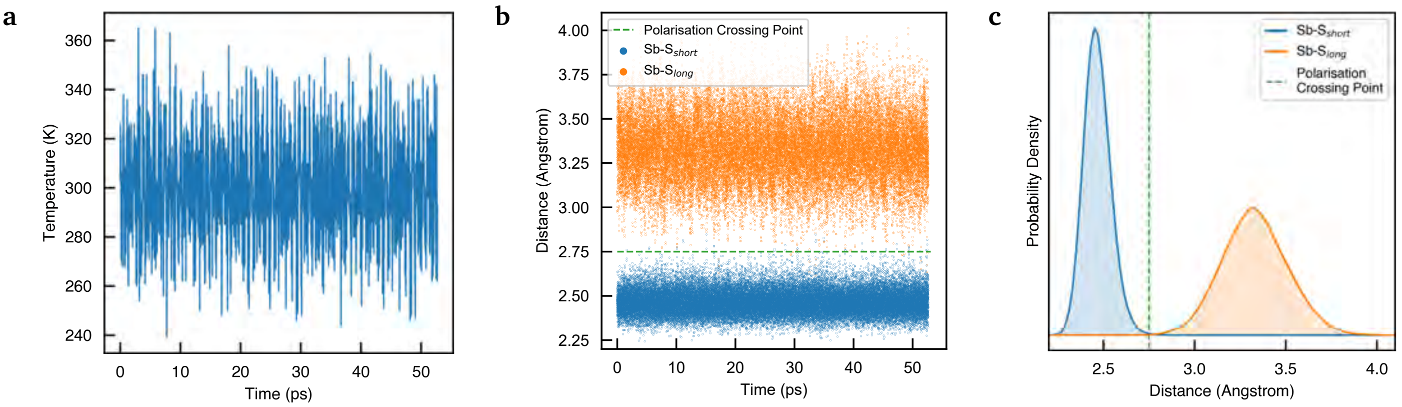

Fig. S26 Variation of supercell temperature **(a)** and Sb–S bond lengths **(b)** as a function of time during the first $T = 300\,\text{K}$ MD run. **c**. Probability densities of Sb–S distances obtained from the first $T = 300\,\text{K}$ MD run.

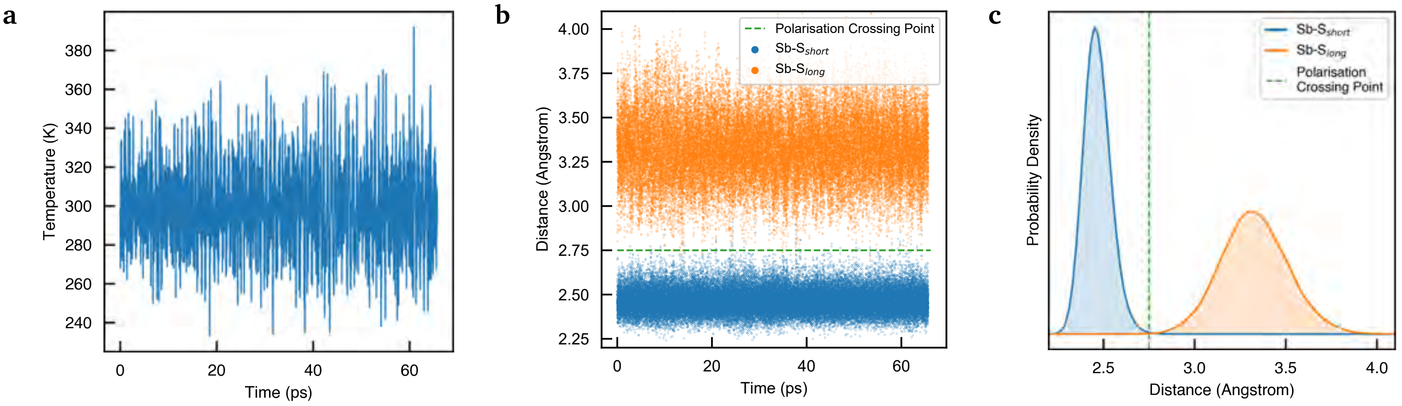

Fig. S27 Variation of supercell temperature **(a)** and Sb–S bond lengths **(b)** as a function of time during the second $T = 300\,\text{K}$ MD run. **c**. Probability densities of Sb–S distances obtained from the second $T = 300\,\text{K}$ MD run.

From these room-temperature simulation runs, we find no switching of the overall polarisation direction, corresponding to switching of the short and long Sb–S bonds, within the simulation timescale. While some temporary switching is observed for individual Sb atoms, as expected, this is rare, momentary ($\sim$fs) and only for single Sb sites (rather than correlated switching for the entire cell). Overall, the Sb atoms are stable in the potential energy well for a given polarisation direction ($Cmc2_1$ configuration), as reflected by their temporal position distributions – further demonstrated by the anisotropic displacement ellipsoids in Fig. S29.

Fig. S28 shows the results of the MD run performed at 227 °C ($T = 500\,\text{K}$).

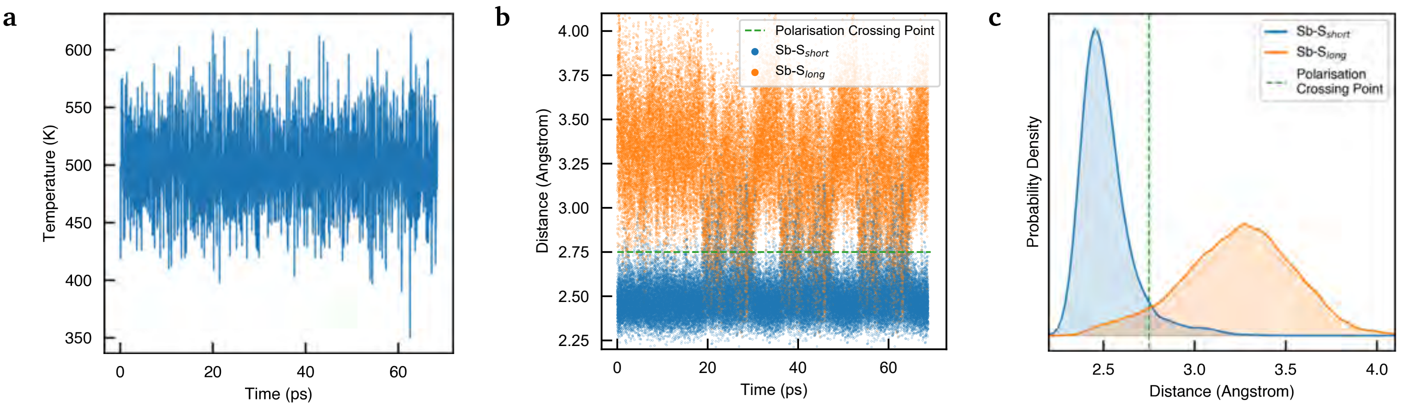

Fig. S28 Variation of supercell temperature **(a)** and Sb–S bond lengths **(b)** as a function of time during the $T = 500\,\text{K}$ MD run. **c**. Probability densities of Sb–S distances obtained from the $T = 500\,\text{K}$ MD run.

In this case, we witness swapping of short and long Sb–S bonds. This switching behaviour is still transient, however, but occurs with significantly longer lifetimes ($\tau \sim$ps) than at $T = 300\,\mathrm{K}$, indicating a significant decrease in both the strength and stability of lattice polarisation at this temperature. Indeed, the material is approaching a phase transition to the higher-symmetry *Cmcm* structure, at which point the 'short' and 'long' Sb–S bonds become equivalent and the radial distribution functions merge to form a single peak (matching that shown in Fig. S25 for *Cmcm*).
From these calculations, we predict that ferroelectric polarisation in $Sn_2SbS_2I_3$ is stable at room temperature, but begins to breakdown as expected at elevated temperatures ($T \geq 500\,\mathrm{K}$).

The anisotropic thermal displacement ellipsoids for $Sn_2SbS_2I_3$, as obtained from these MD simulations, are shown in Fig. S29.

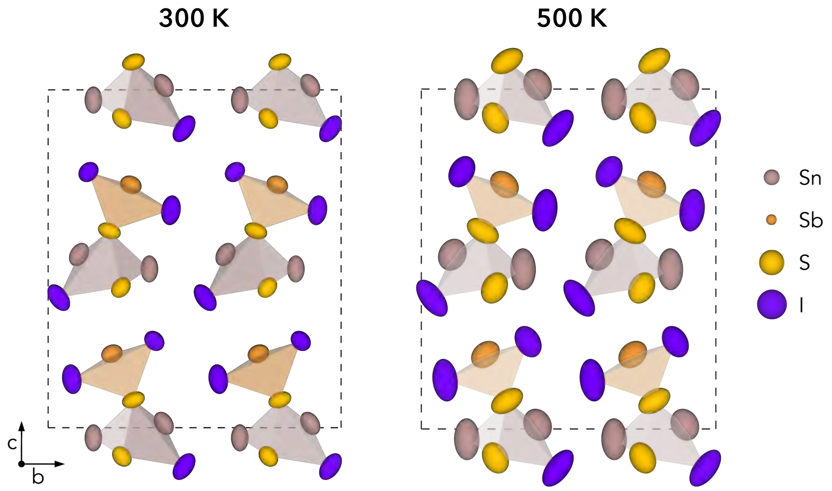

Fig. S29 Averaged thermal displacement ellipsoids for $Sn_2SbS_2I_3$ as obtained from $T = 300\,\mathrm{K}$ **(a)** and $T = 500\,\mathrm{K}$ **(b)** MD simulations, using a 99% confidence interval. Displayed in the conventional orthorhombic unit cell and projected along the *bc* crystal plane.

Crystallographic Information Files (CIFs) with the thermal displacement parameters are provided as supplementary information and on the Zenodo repository.

Table S8 Averaged isotropic ($B = 8\pi^2\overline{U_{jj}}$) and anisotropic ($U_{xx}; \{x = 1,2,3\}$) thermal displacement parameters for $Sn_2SbS_2I_3$ as obtained from $T = 300\,K$ MD simulations. Errors given relative to the room temperature ($T = 293\,K$) experimental values [S9,S11]

Species	$B/Å^2$	U_{11}	U_{22}	U_{33}
Sn	1.9	0.020	0.019	0.033
Sb	1.6	0.020	0.024	0.018
S	1.6	0.021	0.024	0.019
I	2.0	0.020	0.022	0.034
**Experiment:**	T =293 K			
Sn	2.3	0.030	0.033	0.027
Sb	1.9	0.028	0.031	0.030
S	1.1	0.024	0.010	0.010
I	1.4	0.018	0.019	0.017
**Experiment:**	T =173 K			
Sn	1.8	0.022	0.030	0.019
Sb	1.6	0.019	0.022	0.027
S	0.8	0.020	0.007	0.008
I	1.0	0.013	0.014	0.012

It should be noted that the atomic displacement parameters in Table S8 are given with respect to the average positions of the ions during the MD simulation, whereas experimental values were obtained from XRD measurements *assuming a Cmcm crystal structure*, and so may not be directly comparable. That said, the sum of the averaged isotropic displacement parameters (*i.e.* mean-squared displacements) at $T = 300\,K$, $\sum B = 7.1$, matches well with the experimental report $\sum B_{Exp.} = 6.7$ at $T = 293\,K$.

S9 Experimental Crystal Structure Validation

We propose that the experimental characterisation techniques of

- Low-Temperature X-Ray Diffraction (LT XRD)
- Extended X-ray Absorption Fine Structure (EXAFS)
- Neutron Diffraction
- Temperature-Dependent Vibrational Spectroscopy (Raman/Infrared)

or a combination thereof could be employed to avoid macroscopic averaging and verify the microscopic $Cmc2_1$ crystal structure for $Sn_2SbS_2I_3$.

Additionally, inversion symmetry breaking and lattice polarisation could potentially be confirmed through measurements of a non-linear optical (NLO) response, such as second harmonic generation (SHG). Indeed, SHG measurements have been well-established as a useful technique for demonstrating non-centrosymmetry in crystals.[S108,S109]

Notes and references

1 Y.-T. Huang, S. R. Kavanagh, D. O. Scanlon, A. Walsh and R. L. Z. Hoye, *Nanotechnology*, 2021, **32**, 132004.
2 M. Buchanan, *Nature Physics*, 2020, **16**, 996–996.
3 A. M. Ganose, C. N. Savory and D. O. Scanlon, *Chemical Communications*, 2017, **53**, 20–44.
4 R. Nie, K. S. Lee, M. Hu, M. J. Paik and S. I. Seok, *Matter*, 2020, S2590238520304471.
5 R. Nie, R. R. Sumukam, S. H. Reddy, M. Banavoth and S. I. Seok, *Energy & Environmental Science*, 2020, **13**, 2363–2385.
6 R. Nie, M. Hu, A. M. Risqi, Z. Li and S. I. Seok, *Advanced Science*, 2021, **8**, 2003172.
7 A. Zakutayev, J. D. Major, X. Hao, A. Walsh, J. Tang, T. K. Todorov, L. H. Wong and E. Saucedo, *Journal of Physics: Energy*, 2021.
8 Y. C. Choi and K.-W. Jung, *Nanomaterials*, 2020, **10**, 2284.
9 J. Olivier-Fourcade, J. C. Jumas, M. Maurin and E. Philippot, *Zeitschrift für anorganische und allgemeine Chemie*, 1980, **468**, 91–98.
10 A. Ibanez, J.-C. Jumas, J. Olivier-Fourcade and E. Philippot, *Journal of Solid State Chemistry*, 1984, **55**, 83–91.

11 V. Dolgikh, *Izvestiya Akademii Nauk SSSR, Neorganicheskie Materialy*, 1985, **21**, 1211–1214.
12 V. I. Starosta, J. Kroutil and L. Beneš, *Crystal Research and Technology*, 1990, **25**, 1439–1442.
13 A. Kojima, K. Teshima, Y. Shirai and T. Miyasaka, *Journal of the American Chemical Society*, 2009, **131**, 6050–6051.
14 G. Kresse and J. Hafner, *Physical Review B*, 1993, **47**, 558–561.
15 G. Kresse and J. Hafner, *Physical Review B*, 1994, **49**, 14251–14269.
16 G. Kresse and J. Furthmüller, *Computational Materials Science*, 1996, **6**, 15–50.
17 G. Kresse and J. Furthmüller, *Physical Review B - Condensed Matter and Materials Physics*, 1996, **54**, 11169–11186.
18 J. Klimeš, D. R. Bowler and A. Michaelides, *Physical Review B*, 2011, **83**, 195131.
19 A. Lozano, B. Escribano, E. Akhmatskaya and J. Carrasco, *Physical Chemistry Chemical Physics*, 2017, **19**, 10133–10139.
20 S. A. Tawfik, T. Gould, C. Stampfl and M. J. Ford, *Physical Review Materials*, 2018, **2**, 034005.
21 F. Tran, L. Kalantari, B. Traoré, X. Rocquefelte and P. Blaha, *Physical Review Materials*, 2019, **3**, 063602.
22 S. Jaśkaniec, S. R. Kavanagh, J. Coelho, S. Ryan, C. Hobbs, A. Walsh, D. O. Scanlon and V. Nicolosi, *npj 2D Materials and Applications*, 2021, **5**, 1–9.
23 J. Heyd, G. E. Scuseria and M. Ernzerhof, *The Journal of Chemical Physics*, 2003, **118**, 8207–8215.
24 J. Harl, L. Schimka and G. Kresse, *Physical Review B*, 2010, **81**, 115126.
25 M. Kaltak, J. Klimeš and G. Kresse, *Journal of Chemical Theory and Computation*, 2014, **10**, 2498–2507.
26 P. Borlido, J. Schmidt, A. W. Huran, F. Tran, M. A. L. Marques and S. Botti, *npj Computational Materials*, 2020, **6**, 1–17.
27 A. V. Krukau, O. A. Vydrov, A. F. Izmaylov and G. E. Scuseria, *The Journal of Chemical Physics*, 2006, **125**, 224106.
28 A. Jain, S. P. Ong, G. Hautier, W. Chen, W. D. Richards, S. Dacek, S. Cholia, D. Gunter, D. Skinner, G. Ceder and K. a. Persson, *APL Materials*, 2013, **1**, 011002.
29 K. T. Butler, J. M. Frost and A. Walsh, *Energy & Environmental Science*, 2015, **8**, 838–848.
30 K. T. Butler, S. McKechnie, P. Azarhoosh, M. van Schilfgaarde, D. O. Scanlon and A. Walsh, *Applied Physics Letters*, 2016, **108**, 112103.
31 A. M. Ganose, K. T. Butler, A. Walsh and D. O. Scanlon, *Journal of Materials Chemistry A*, 2016, **4**, 2060–2068.
32 H. Shi, W. Ming and M.-H. Du, *Physical Review B*, 2016, **93**, 104108.
33 A. Walsh, D. J. Payne, R. G. Egdell and G. W. Watson, *Chemical Society Reviews*, 2011, **40**, 4455–4463.
34 C. N. Savory and D. O. Scanlon, *Journal of Materials Chemistry A*, 2019, **7**, 10739–10744.
35 C. H. Don, H. Shiel, T. D. C. Hobson, C. N. Savory, J. E. N. Swallow, M. J. Smiles, L. A. H. Jones, T. J. Featherstone, P. K. Thakur, T.-L. Lee, K. Durose, J. D. Major, V. R. Dhanak, D. O. Scanlon and T. D. Veal, *Journal of Materials Chemistry C*, 2020, **8**, 12615–12622.
36 G. Rhodes, *Crystallography Made Crystal Clear: A Guide for Users of Macromolecular Models*, Elsevier, Amsterdam, 3rd edn, 2006.
37 K. B. Spooner, M. Einhorn and D. W. Davies, *GitHub:SMTG_UCL/ThermoPlotter*, 2020.
38 R. L. Z. Hoye, P. Schulz, L. T. Schelhas, A. M. Holder, K. H. Stone, J. D. Perkins, D. Vigil-Fowler, S. Siol, D. O. Scanlon, A. Zakutayev, A. Walsh, I. C. Smith, B. C. Melot, R. C. Kurchin, Y. Wang, J. Shi, F. C. Marques, J. J. Berry, W. Tumas, S. Lany, V. Stevanović, M. F. Toney and T. Buonassisi, *Chemistry of Materials*, 2017, **29**, 1964–1988.
39 J. Harl and G. Kresse, *Physical Review Letters*, 2009, **103**, 056401.
40 H. T. Stokes, D. M. Hatch and B. J. Campbell, *ISODISTORT*, ISOTROPY Software Suite.
41 B. J. Campbell, H. T. Stokes, D. E. Tanner and D. M. Hatch, *Journal of Applied Crystallography*, 2006, **39**, 607–614.
42 R. D. King-Smith and D. Vanderbilt, *Physical Review B*, 1993, **47**, 1651–1654.
43 H. H. Wieder, *Physical Review*, 1955, **99**, 1161–1165.
44 A. Stroppa, C. Quarti, F. De Angelis and S. Picozzi, *The Journal of Physical Chemistry Letters*, 2015, **6**, 2223–2231.
45 A. A. Grekov, A. I. Rodin and V. M. Fridkin, *Applied Physics Letters*, 1969, **14**, 119–120.
46 J. M. Frost, K. T. Butler, F. Brivio, C. H. Hendon, M. van Schilfgaarde and A. Walsh, *Nano Letters*, 2014, **14**, 2584–2590.
47 N. A. Spaldin, *Journal of Solid State Chemistry*, 2012, **195**, 2–10.
48 D. H. Fabini, G. Laurita, J. S. Bechtel, C. C. Stoumpos, H. A. Evans, A. G. Kontos, Y. S. Raptis, P. Falaras, A. Van der Ven, M. G. Kanatzidis and R. Seshadri, *Journal of the American Chemical Society*, 2016, **138**, 11820–11832.
49 J. Brgoch, A. J. Lehner, M. Chabinyc and R. Seshadri, *The Journal of Physical Chemistry C*, 2014, **118**, 27721–27727.
50 H. Jónsson, G. Mills and K. W. Jacobsen, *Classical and Quantum Dynamics in Condensed Phase Simulations*, World Scientific, 1998, pp. 385–404.
51 S. Song, H. M. Jang, N.-S. Lee, J. Y. Son, R. Gupta, A. Garg, J. Ratanapreechachai and J. F. Scott, *NPG Asia Materials*, 2016, **8**, e242–e242.
52 B. Chen, J. Shi, X. Zheng, Y. Zhou, K. Zhu and S. Priya, *Journal of Materials Chemistry A*, 2015, **3**, 7699–7705.
53 I. Grinberg, D. V. West, M. Torres, G. Gou, D. M. Stein, L. Wu, G. Chen, E. M. Gallo, A. R. Akbashev, P. K. Davies, J. E. Spanier and A. M. Rappe, *Nature*, 2013, **503**, 509–512.
54 P. S. Brody, *Ferroelectrics*, 1975.
55 T. S. Sherkar and L. J. A. Koster, *Physical Chemistry Chemical Physics*, 2016, **18**, 331–338.
56 F. Zheng, H. Takenaka, F. Wang, N. Z. Koocher and A. M. Rappe, *The Journal of Physical Chemistry Letters*, 2015, **6**, 31–37.
57 S. Picozzi, *Frontiers in Physics*, 2014, **2**, 10.
58 C. W. Myung, J. Yun, G. Lee and K. S. Kim, *Advanced Energy Materials*, 2018, **8**, 1702898.
59 A. M. Ganose, A. J. Jackson and D. O. Scanlon, *Journal of Open Source Software*, 2018, **3**, 717.
60 L. Yu and A. Zunger, *Physical Review Letters*, 2012, **108**, 068701.
61 B. Blank, T. Kirchartz, S. Lany and U. Rau, *Physical Review Applied*, 2017, **8**, 024032.
62 W. Shockley and H. J. Queisser, *Journal of Applied Physics*, 1961, **32**, 510–519.
63 D. R. Lide, *CRC Handbook of Chemistry and Physics*, CRC Press, Florida, Eighty-fourth edn, 2003.
64 Z. Li, S. R. Kavanagh, M. Napari, R. G. Palgrave, M. Abdi-Jalebi, Z. Andaji-Garmaroudi, D. W. Davies, M. Laitinen, J. Julin, M. A. Isaacs, R. H. Friend, D. O. Scanlon, A. Walsh and R. L. Z. Hoye, *Journal of Materials Chemistry A*, 2020, **8**, 21780–21788.
65 S. R. Kavanagh, A. Walsh and D. O. Scanlon, *ACS Energy Letters*, 2021, **6**, 1392–1398.
66 S. Kim, J. A. Márquez, T. Unold and A. Walsh, *Energy & Environmental Science*, 2020, 1481–1491.
67 C. Freysoldt, B. Grabowski, T. Hickel, J. Neugebauer, G. Kresse, A. Janotti and C. G. Van de Walle, *Reviews of Modern Physics*, 2014, **86**, 253–305.
68 A. Walsh and A. Zunger, *Nature Materials*, 2017, **16**, 964–967.
69 R. C. Kurchin, P. Gorai, T. Buonassisi and V. Stevanović, *Chemistry of Materials*, 2018, **30**, 5583–5592.
70 V. L. Deringer and R. Dronskowski, *Comprehensive Inorganic Chemistry II (Second Edition)*, Elsevier, Amsterdam, 2013, pp. 59–87.
71 R. Dronskowski and P. E. Bloechl, *The Journal of Physical Chemistry*, 1993, **97**, 8617–8624.
72 K. T. Butler, J. M. Frost and A. Walsh, *Materials Horizons*, 2015, **2**, 228–231.
73 Z. Zhao, F. Gu, Y. Li, W. Sun, S. Ye, H. Rao, Z. Liu, Z. Bian and C. Huang, *Advanced Science*, 2017, **4**, 1700204.
74 Y. Zhou, L. Wang, S. Chen, S. Qin, X. Liu, J. Chen, D.-J. Xue, M. Luo, Y. Cao, Y. Cheng, E. H. Sargent and J. Tang, *Nature Photonics*, 2015, **9**, 409–415.
75 S. P. Huber, S. Zoupanos, M. Uhrin, L. Talirz, L. Kahle, R. Häuselmann, D. Gresch, T. Müller, A. V. Yakutovich, C. W. Andersen, F. F. Ramirez, C. S. Adorf, F. Gargiulo, S. Kumbhar, E. Passaro, C. Johnston, A. Merkys, A. Cepellotti, N. Mounet, N. Marzari, B. Kozinsky and G. Pizzi, *Scientific Data*, 2020, **7**, 300.
76 P. E. Blöchl, *Physical Review B*, 1994, **50**, 17953–17979.
77 A. J. Jackson, A. M. Ganose, A. Regoutz, R. G. Egdell and D. O. Scanlon, *Journal of Open Source Software*, 2018, **3**, 773.
78 M. Iraola, J. L. Mañes, B. Bradlyn, T. Neupert, M. G. Vergniory and S. S. Tsirkin, *arXiv:2009.01764 [cond-mat, physics:physics]*, 2020.
79 L. Elcoro, B. Bradlyn, Z. Wang, M. G. Vergniory, J. Cano, C. Felser, B. A. Bernevig, D. Orobengoa, G. de la Flor and M. I. Aroyo, *Journal of Applied Crystallography*, 2017, **50**, 1457–1477.
80 Q. Zheng, *GitHub:QijingZheng/VASP_TDM*, 2020.
81 K. T. Butler, C. H. Hendon and A. Walsh, *Journal of the American Chemical Society*, 2014, **136**, 2703–2706.

82 K. Brlec, D. W. Davies and D. O. Scanlon, *Journal of Open Source Software*, 2021, **6**, 3171.
83 A. Ganose, *GitHub:Utf/Bapt*, 2020.
84 M. Gajdoš, K. Hummer, G. Kresse, J. Furthmüller and F. Bechstedt, *Physical Review B*, 2006, **73**, 045112.
85 J. P. Perdew, A. Ruzsinszky, G. I. Csonka, O. A. Vydrov, G. E. Scuseria, L. A. Constantin, X. Zhou and K. Burke, *Physical Review Letters*, 2008, **100**, 136406.
86 K. Parlinski, Z. Q. Li and Y. Kawazoe, *Physical Review Letters*, 1997, **78**, 4063–4066.
87 A. Togo and I. Tanaka, *Scripta Materialia*, 2015, **108**, 1–5.
88 W. G. Hoover, *Physical Review A*, 1985, **31**, 1695–1697.
89 S. Nosé, *The Journal of Chemical Physics*, 1984, **81**, 511–519.
90 A. Tkatchenko and M. Scheffler, *Physical Review Letters*, 2009, **102**, 073005.
91 F. Birch, *Physical Review*, 1947, **71**, 809–824.
92 A. Stukowski, *Modelling and Simulation in Materials Science and Engineering*, 2009, **18**, 015012.
93 B. J. Morgan, *Vasppy*, zenodo.org/record/4460130, 2021.
94 A. Carreras, A. Togo and I. Tanaka, *Computer Physics Communications*, 2017, **221**, 221–234.
95 S. P. Ong, W. D. Richards, A. Jain, G. Hautier, M. Kocher, S. Cholia, D. Gunter, V. L. Chevrier, K. A. Persson and G. Ceder, *Computational Materials Science*, 2013, **68**, 314–319.
96 R. D. Shannon, *Acta Crystallographica Section A*, 1976, **32**, 751–767.
97 T. Bučko, S. Lebègue, J. G. Ángyán and J. Hafner, *The Journal of Chemical Physics*, 2014, **141**, 034114.
98 B. Silvi and A. Savin, *Nature*, 1994, **371**, 683–686.
99 Y. Gassenbauer and A. Klein, *The Journal of Physical Chemistry B*, 2006, **110**, 4793–4801.
100 M. G. Helander, M. T. Greiner, Z. B. Wang, W. M. Tang and Z. H. Lu, *Journal of Vacuum Science & Technology A*, 2011, **29**, 011019.
101 F. Brivio, K. T. Butler, A. Walsh and M. van Schilfgaarde, *Physical Review B*, 2014, **89**, 155204.
102 T. J. Whittles, L. A. Burton, J. M. Skelton, A. Walsh, T. D. Veal and V. R. Dhanak, *Chemistry of Materials*, 2016, **28**, 3718–3726.
103 W. Setyawan and S. Curtarolo, *Computational Materials Science*, 2010, **49**, 299–312.
104 Y. Hinuma, G. Pizzi, Y. Kumagai, F. Oba and I. Tanaka, *Computational Materials Science*, 2017, **128**, 140–184.
105 P. Y. Yu and M. Cardona, *Fundamentals of Semiconductors*, Springer Berlin Heidelberg, Berlin, Heidelberg, 4th edn, 2010.
106 V. Wang, N. Xu, J. C. Liu, G. Tang and W.-T. Geng, *arXiv:1908.08269 [cond-mat]*, 2020.
107 J. M. Munro, H. Akamatsu, H. Padmanabhan, V. S. Liu, Y. Shi, L.-Q. Chen, B. K. VanLeeuwen, I. Dabo and V. Gopalan, *Physical Review B*, 2018, **98**, 085107.
108 K. M. Ok, E. O. Chi and P. S. Halasyamani, *Chemical Society Reviews*, 2006, **35**, 710–717.
109 A. Coda and F. Pandarese, *Journal of Applied Crystallography*, 1976, **9**, 193–198.
